# Genomic rearrangements and evolutionary changes in 3D chromatin topologies in the cotton tribe (*Gossypieae*)

**DOI:** 10.1186/s12915-023-01560-y

**Published:** 2023-03-20

**Authors:** Xiaochong Li, Jinbin Wang, Yanan Yu, Guo Li, Jinpeng Wang, Changping Li, Zixian Zeng, Ning Li, Zhibin Zhang, Qianli Dong, Yiyang Yu, Xiaofei Wang, Tianya Wang, Corrinne E. Grover, Bin Wang, Bao Liu, Jonathan F. Wendel, Lei Gong

**Affiliations:** 1 Key Laboratory of Molecular Epigenetics of the Ministry of Education (MOE), Northeast Normal University, Changchun, 130024 China; 2 School of Life Sciences, and Center for Genomics and Computational Biology, North China University of Science and Technology, Tangshan, 063000 Hebei China; 3 Department of Biological Science, College of Life Science, Sichuan Normal University, Chengdu, 610101 China; 4 State Key Laboratory of Plant Genomics and National Center for Plant Gene Research, Institute of Genetics and Developmental Biology, Chinese Academy of Sciences, Beijing, 100101 China; 5 Hainan Yazhou Bay Seed Lab, Sanya, 572025 Hainan China; 6 Department of Ecology, Evolution and Organismal Biology, Iowa State University, Ames, IA USA

**Keywords:** Chromatin architecture, *Gossypieae*, Chromosome rearrangement, Hi-C, Epigenetic modifications

## Abstract

**Background:**

Analysis of the relationship between chromosomal structural variation (synteny breaks) and 3D-chromatin architectural changes among closely related species has the potential to reveal causes and correlates between chromosomal change and chromatin remodeling. Of note, contrary to extensive studies in animal species, the pace and pattern of chromatin architectural changes following the speciation of plants remain unexplored; moreover, there is little exploration of the occurrence of synteny breaks in the context of multiple genome topological hierarchies within the same model species.

**Results:**

Here we used Hi-C and epigenomic analyses to characterize and compare the profiles of hierarchical chromatin architectural features in representative species of the cotton tribe (*Gossypieae*), including *Gossypium arboreum*, *Gossypium raimondii*, and *Gossypioides kirkii*, which differ with respect to chromosome rearrangements. We found that (*i*) overall chromatin architectural territories were preserved in *Gossypioides* and *Gossypium*, which was reflected in their similar intra-chromosomal contact patterns and spatial chromosomal distributions; (*ii*) the non-random preferential occurrence of synteny breaks in A compartment significantly associate with the B-to-A compartment switch in syntenic blocks flanking synteny breaks; (*iii*) synteny changes co-localize with open-chromatin boundaries of topologically associating domains, while TAD stabilization has a greater influence on regulating orthologous expression divergence than do rearrangements; and (*iv*) rearranged chromosome segments largely maintain ancestral *in-cis* interactions.

**Conclusions:**

Our findings provide insights into the non-random occurrence of epigenomic remodeling relative to the genomic landscape and its evolutionary and functional connections to alterations of hierarchical chromatin architecture, on a known evolutionary timescale.

**Supplementary Information:**

The online version contains supplementary material available at 10.1186/s12915-023-01560-y.

## Background

Chromosome karyotypes vary enormously among organisms [1–4], even among closely related species, where chromosomal rearrangements (represented by synteny breaks) are often observed [5–8]. In addition to these linear features, the three-dimensional (3D) organization of chromatin and its interactions play vital roles in cellular processes [9–15]. Recent advances in chromatin conformation capture technologies, especially high-throughput chromosome conformation capture (Hi-C), have enabled the exploration of hierarchical chromatin architectures/topologies and their role in regulating gene expression in plants [11, 16–20] and animals [10, 21–25]. These chromosomal architectural features include chromosomal territories (CTs; discrete space occupied by nuclear chromosomes), A/B compartments (large chromosomal segments displaying similar interaction patterns within the same type, considered as “active” and “inactive” genomic regions, respectively), topologically associated domains (TADs; *cis* interacting domains harboring higher frequency of intra-TAD interaction than among TAD), and fine-scale *in-cis* interactions. At present, relatively little is understood regarding the connections between these higher-order, chromatin-level epigenomic alterations and chromosome rearrangements at the genomic level. Such an understanding might facilitate insight into the functional and perhaps evolutionary consequences of genomic rearrangements accompanying speciation.

Many studies in animals have explored the potential role of genomic rearrangements in remodeling different chromatin architecture, as revealed by Hi-C interaction maps [5, 6, 8, 26–29]. In mammals, for example, at the compartment A/B level, orthologous sub-chromosomal fragments display conserved 3D chromatin architecture, while sex chromosomes (i.e., neo-Y and X chromosome) with extensive rearrangements have pronounced differences in chromatin compartments [8, 29]. At the TAD level, conserved TADs without genomic rearrangements in representative vertebrates (human to mouse comparison) harbor genes with higher expression than those residing in non-conserved TADs with shuffled genomic fragments [30]; the genomic rearrangements that shuffle TADs rarely affect the role of TADs in regulating gene expression within fruit flies [6]. Of note, the pace and pattern of chromatin architectural change following the speciation of plants remain unexplored.

Available studies in animal and several plant models have implicated preferential occurrence of synteny breaks associated with particular chromatin architectural features. Within mosquitoes, synteny breaks preferentially occurred within active euchromatic regions belonging to A compartments [28]. At a finer scale, the co-localization of synteny breaks with TAD boundaries has been consistently identified in chicken, gibbon, fruit fly, and green pepper [5–7, 26]. Notably, there is little exploration of the occurrence of synteny breaks in the context of multiple genome topological hierarchies within the same model species.

Prerequisites for exploring the connection between genomic remodeling and chromatin architectural variation include the existence of a well-established phylogenetic framework among closely related species harboring clear chromosomal rearrangements and high-resolution chromatin interaction maps. In this respect a useful model is the small monophyletic tribe *Gossypieae*, containing the economically important cotton genus (*Gossypium*; 2n = 26) and its closely related East African/Madagascan sister genus (along with *Kokia*) *Gossypioides* (2n = 24). Molecular clock dating based on whole genome data sets has established that *Gossypium* and *Gossypioides* diverged approximately 10 million years ago (MYA) [31]. Following this divergence, a complicated series of chromosomal fission and fusion events occurred in the *Gossypioides* lineage), leading to a dysploid reduction from a haploid complement of *n* = 13 chromosomes to *n* = 12 [30–32]. Within *Gossypium*, a global diversification gave rise to species groups that vary three-fold in genome size while sharing the same chromosome number [33, 34]. Relevant here are the African-Asian *G. arboreum* (A_2_) and the Peruvian *G. raimondii* (D_5_), which differ in several genomic translocations [35] and about two-fold in genome size. In addition to this phylogenetically well-developed framework, chromosome-level genome assemblies have been generated, facilitating the determination of chromatin hierarchies and epigenomic status based on Hi-C and epigenomic sequencing [32, 36–39].

In this study, we adopted Hi-C technology to construct the first high-resolution chromatin interaction map of *Gossypioides* (represented here by *G. kirkii*) and significantly improved the resolution of Hi-C interaction maps of *G. arboreum* and *G. raimondii*. We identified their chromosome rearrangement events represented by synteny breaks. Based on these data, we explored genomic remodeling among chromatin positions at the 3D chromatin level and investigated the relationships between genomic change and different aspects of chromatin architecture. Our findings provide insights into the non-random occurrence of epigenomic remodeling relative to the genomic landscape and its evolutionary and functional connections to alterations of hierarchical chromatin architecture, on a known evolutionary timescale.

## Results

### Identification of genomic rearrangements in the two genera *Gossypium* and *Gossypioides*

**Fig. 1:**
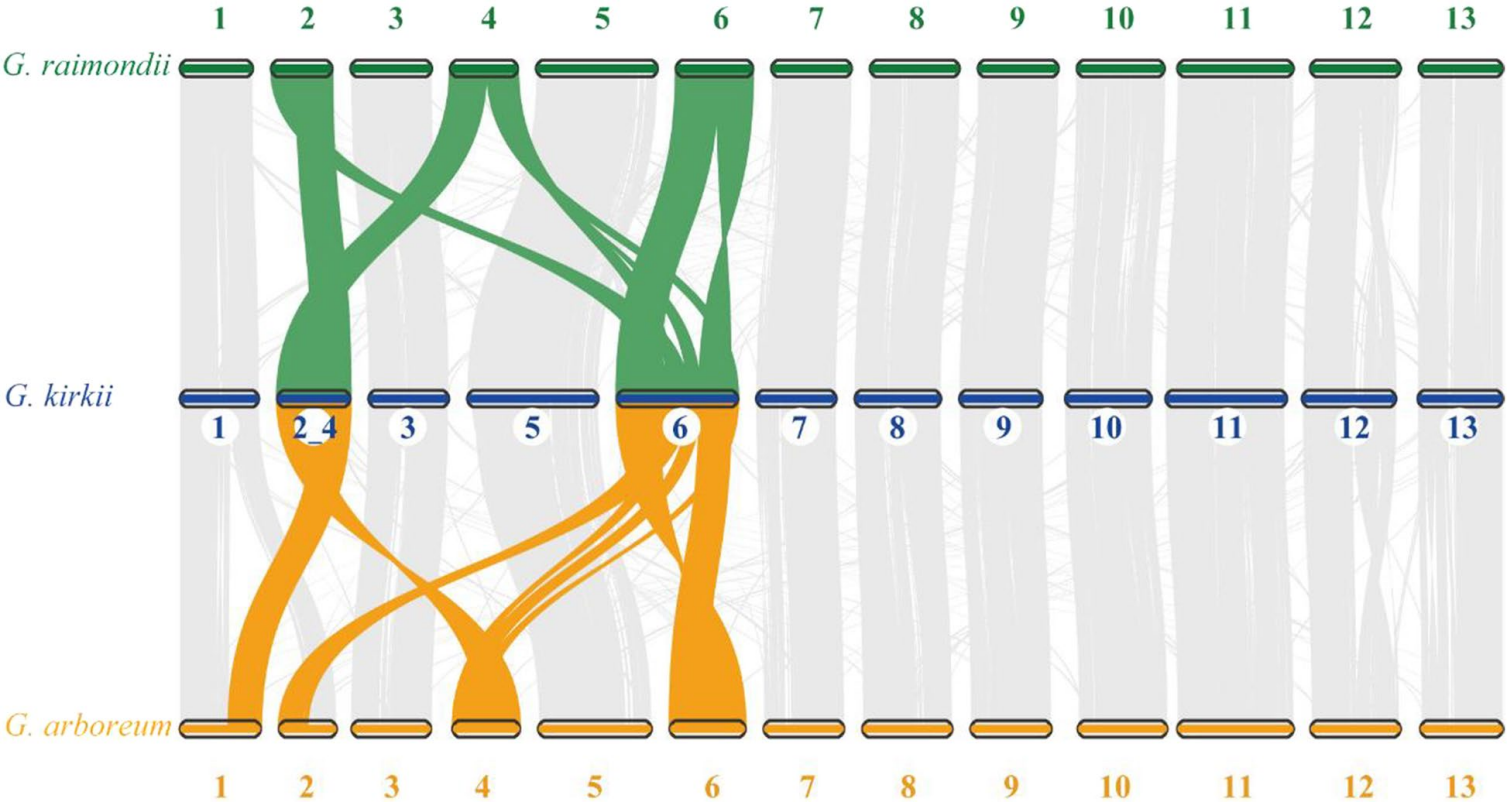
Chromosomal synteny among *G. arboreum* (A_2_), *Gossypioides kirkii*, and *G. raimondii* (D_5_) with chromosome names denoted. Yellow and green lines indicate, respectively, the large rearrangements between *G. kirkii vs. G. arboreum* and *G. kirkii vs. G. raimondii*. Gray lines denote the other syntenic blocks in *G. kirkii* in comparison to their counterparts in *G. arboreum* and *G. raimondii*

Genomic or chromosomal rearrangements, represented by synteny breaks, are commonly identified between closely related plant species [40–44]. In our case, after speciation about 10 MYA (million years ago), *Gossypioides kirkii* (2n = 24, *n* = 12) and *Gossypium* species (2n = 26, *n* = 13; including *G. arboreum* and *G. raimondii*) accumulated synteny breaks via chromosomal rearrangements [31, 32]. Based upon strict filtering criteria (Additional file 1: Fig. S1; Methods), we characterized a total of 1452 and 964 synteny breaks in *G. kirkii* vs. *G. arboreum* and *G. kirkii* vs. *G. raimondii*, respectively (Fig. 1). Similar to the previous findings [32], further syntenic analysis revealed that one entire arm of ancestral Chr02 in the common ancestor of *G. arboreum* and *G. raimondii* merged with an entire arm of ancestral Chr04 to form the major segment of a single chromosome KI_2_4 in *G. kirkii* (Fig. 1, Additional file 1: Fig. S2a, b); the remaining chromosome arms were broken and inserted into chromosome KI_06, which partially explains the chromosomal number difference between the genera *Gossypioides* and *Gossypium* (Fig. 1, Additional file 1: Fig. S2a, b). Additionally, the previously recognized chromosomal translocation between Chr01 and Chr02 segments was identified in *G. arboreum* (Fig. 1, Additional file 1: Fig. S2c) [35, 38]. Finally, a chromosomal inversion was identified in Chr02 of *G. arboreum* (Additional file 1: Fig. S2c; see details in the last section of the “Results” section). These clearly demarcated synteny breaks induced by genomic rearrangements allow us to explore their relationship to 3D chromatin architectural features.

Firstly, we characterized the distribution of synteny breaks across three genomes. This analysis shows that synteny breaks are highly concentrated on both chromosomal arms (Additional file 1: Fig. S3a). To demonstrate the epigenetic correlations of the identified synteny breaks, we examined the status of numerous epigenetic modifications (histone marks and DNA methylation by ChIP-seq and BS-seq; Methods) near synteny breaks (± 20 kb) within sampled *Gossypioides* and *Gossypium* species (Additional file 1: Fig. S3b). We found that synteny breaks were enriched for H3K4me3 and H3K27me3, but depleted for H3K9me2 and DNA methylation in all three context sequences (CG, CHG, and CHH, where H is any base except G). We further characterized the distribution of transposable elements (Gypsy and Copia) relative to synteny breaks and found decreasing TE insertion frequency in adjacency to the synteny breaks (Additional file 1: Fig. S3b). These results implicate that synteny breaks likely occurred in open chromatin in the respective species. Finally, gene ontology (GO) analyses have been carried out for genes adjacent to synteny breaks. The results show that these genes were overrepresented in DNA-binding transcription factor activity, AT DNA binding, sequence-specific DNA binding, and hydrolase activity (Additional file 1: Fig. S3c).

### Preserved overall chromosomal contact patterns in *Gossypioides* and *Gossypium*

To assess whether genome rearrangements were connected by either cause or effect to chromatin architectural features, we generated high-resolution, Hi-C interaction maps based on in situ Hi-C experiments with two biological replicates of young leaves (Methods). Briefly, after stringent filtering, we obtained a total of ~ 1312, ~ 3738, and ~ 3151 million valid paired-end (PE) reads in *G. kirkii*, *G. arboreum*, and *G. raimondii* (Additional file 1: Tables S1–S3). Based on consistency between replicates (Additional file 1: Fig. S4a), we combined Hi-C data from different replicates to construct high-resolution contact maps for each species. Notably, our chromatin interaction map of *G. kirkii* reached 1-kb resolution, and also we achieved significant improvements in resolution relative to earlier data for both *G. arboreum* and *G. raimondii* (an increase of resolution from 20 to 5 kb and from 10 to 1 kb, respectively; Additional file 1: Fig. S4b) [38]. These high-resolution Hi-C contact maps enabled to identify hierarchical chromatin architectural features, including chromosomal territories, A/B compartments, and topologically associated domains.

Potential association between genetic rearrangements and chromosomal territories (abbreviated as CTs) were analyzed for orthologous chromosomes not involved in inter-chromosomal rearrangements [32]. We first investigated the CTs of chromosomes in *G. kirkii*, *G. arboreum*, and *G. raimondii* by visualizing their genome-wide Hi-C matrix at 100-kb resolution (Additional file 1: Fig. S5). Consistent with other plant species, CTs feature stronger intra-chromosomal interactions with significantly weaker inter-chromosomal interactions visible in the contact map of each species (Additional file 1: Fig. S5). We next assessed CTs by estimating the whole-chromosomal interactions between chromosome pairs based on the iterative correction and eigenvector (ICE)-corrected Hi-C interaction matrix in each species (Additional file 1: Fig. S6). We found that, for the most part, relative chromosomal position and chromosomal space remained generally conserved among the three *Gossypioides* and *Gossypium* species studied, which is especially obvious for the visually similar chromosomal distribution in *G. kirkii* and *G. raimondii* (Additional file 1: Fig. S6, Table S4). We speculate that some genomic or chromosomal feature(s) of *G. arboreum* are responsible for the difference in CTs from those in *G. kirkii* and *G. raimondii*; in this respect, we highlight the much greater difference in genome size and TE content between the larger *G. arboreum* genome and the other two species [31, 32, 36, 38, 45–47]. Overall, synteny breaks that accumulated during the divergence of these three species appeared to have limited effects on overall chromatin architectural CTs, in that intra-chromosomal contact patterns and spatial distributions of chromosomes are mostly preserved.

### Compartment switching/transitions associated with genomic rearrangements

Based on principal component analysis (PCA) for the Hi-C contact matrix, chromosomes may be divided into so-called A and B compartments, corresponding to active and inactive genome regions [10]. We explored the relationships between rearrangements and the reconstruction of compartments in each species. Accordingly, we first identified the A/B compartments in all three species at a 50-kb resolution (Methods). Congruent with compartment patterns in *Gossypium* species reported previously [38, 39], the A compartments localized primarily to chromosome arms, which have higher gene density, a lower DNA methylation level, and more active histone modification (H3K4me3), while B compartments localized mostly to centromeric and pericentromeric regions, which harbor fewer genes, a higher DNA methylation level, and more inactive histone modification (H3K9me2) in all three species (Additional file 1: Fig. S7a). In line with differences in genome size due to distinct TE contents [31, 32, 36, 38, 45–47], the larger *G. arboreum* genome had more inactive B compartments (~ 53.3% of the genome) and fewer active A compartments (~ 46.7%) than those of smaller *G. kirkii* and *G. raimondii* genomes (~ 52.8% and 47.2%, and ~ 51.4% and 48.6%, respectively; Additional file 1: Fig. S7b).

**Fig. 2:**
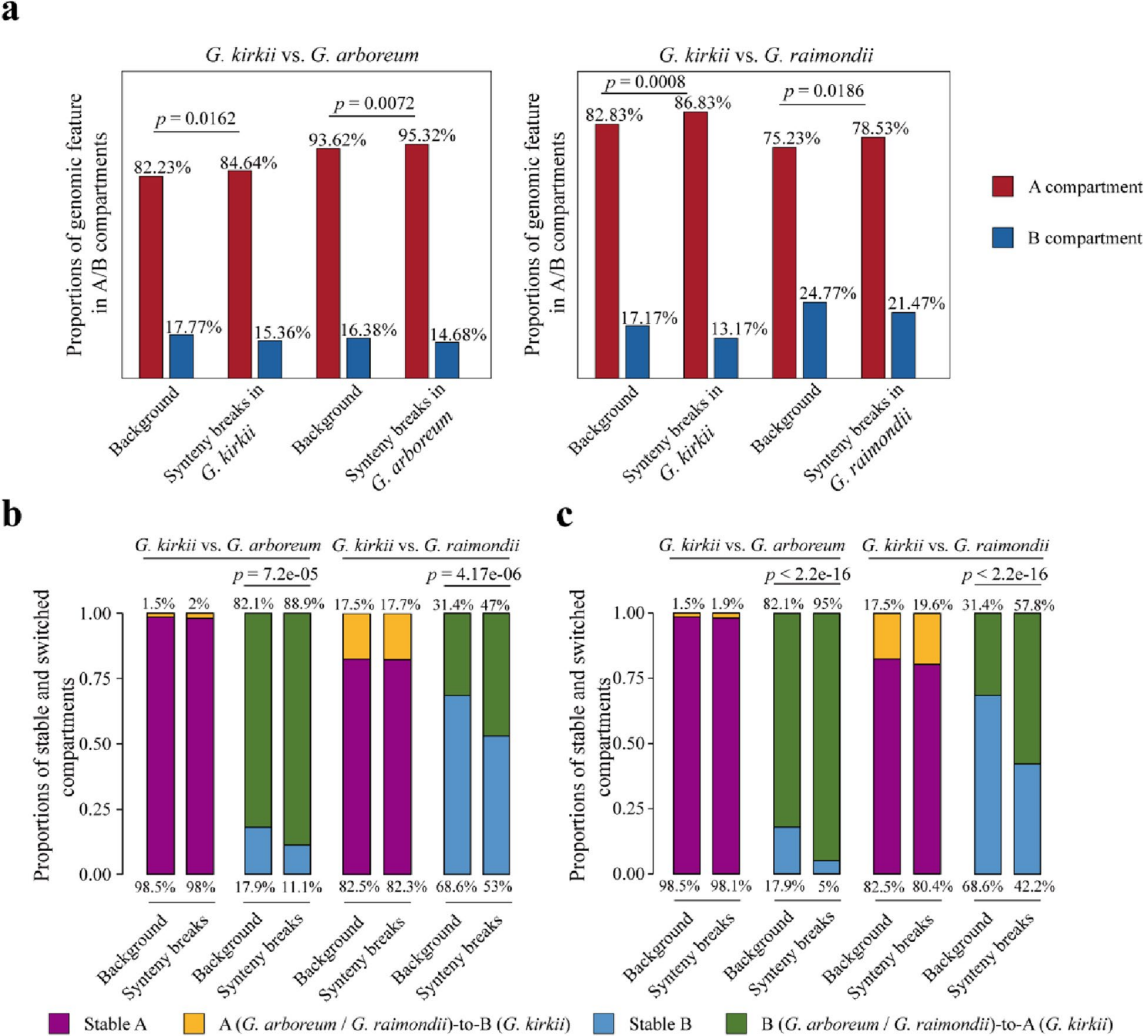
The distribution of synteny breaks relative to A/B compartments in *G. kirkii* and their association with adjacent compartment switches.** a** Proportions of synteny breaks (as revealed by comparisons of *G. kirkii vs. G. arboreum* (left) and *G. kirkii vs. G. raimondii* (right) and other syntenic genes (background) identified in A and B compartments are summarized and compared. **b** Proportions of stable (stable A and stable B) and switched compartments (A to B and B to A) identified in chromatin regions adjacent to respective synteny breaks (± 10 kb) and other syntenic gene regions (background) in *G. kirkii* are summarized and compared. **c** Proportions of stable (stable A and stable B) and switched compartments (A to B and B to A) identified in chromatin regions adjacent to respective synteny breaks (± 10 kb) and other syntenic gene regions (background) in KI_2_4 and KI_06 (with inter-chromosomal chromosomal re-arrangements) of *G. kirkii* are summarized and compared. All statistical significances were calculated using Fisher exact’s test

We hypothesized that synteny breaks are not randomly distributed relative to A/B compartmentalization in the genomes. To test this hypothesis, we analyzed the distribution of synteny breaks relative to the A/B compartments (Fig. 2a). As hypothesized, synteny breaks are significantly enriched in A compartment genomic regions in each species (Fig. 2a). We postulate that this non-random genomic distribution of synteny breaks is causally connected to their more open chromatin state (A compartments) and perhaps to a resulting higher chromatin fragility (relative to B compartments), as in animal species [28, 48].

In comparisons between the two *Gossypium* species (*G. arboreum* and *G. raimondii*) and *G. kirkii*, we identified 2572 and 709 A-to-B and 399 and 1953 B-to-A compartment switches/transitions, respectively. Furthermore, we characterized and compared the DNA methylation in all three context sequences (CG, CHG, and CHH) and histone modifications (H3K4me3, H3K27me3, and H3K9me2) around the stable (in stable compartment regions) and switched genes (in switched compartments) within respective species. As shown (Additional file 1: Fig. S8), as for the regulatory ± 2 kb regions of respective genes, switched genes harbored higher levels of DNA methylation (CG, CHG, and CHH) and H3K9me2 than those stable genes; intriguingly, the H3K4me3 levels of those switched genes were lower than latter stable genes. This result suggests that genes involved in compartment switch harbored featured epigenetic marks.

To investigate whether genomic rearrangement is associated with A/B compartment switching/transitions, we characterized chromatin compartment alterations of different syntenic regions flanking the identified synteny breaks. In paired comparisons, there were significantly larger fractions of syntenic genes adjacent to synteny breaks exhibiting a B to A switch compared with other syntenic regions, as observed for regions flanking chromosomal fission and fusion sites in chromosome KI_2_4 and KI_06 (Fig. 2b, c, Additional file 1: Fig. S9). Together, these observations suggest that genetic rearrangements may have a prominent impact on the compartment status in syntenic blocks flanking synteny breaks.

### Synteny breaks colocalize with open-chromatin boundaries of TADs

At the sub-megabase scale, chromatin can be folded into subtle topologically associated domains (abbreviated as TADs), which are self-interacting genomic regions [22, 49, 50]. Notably, unlike animals, TADs are formed not in a side-by-side manner in plants [51]. Relative to the known co-localization of synteny breaks with TAD boundaries in animal species (choice made between either TAD boundaries or interior bodies) [5, 6, 26, 27], the relationship between genomic rearrangements and TADs remains unclear in plants [7].

**Fig. 3:**
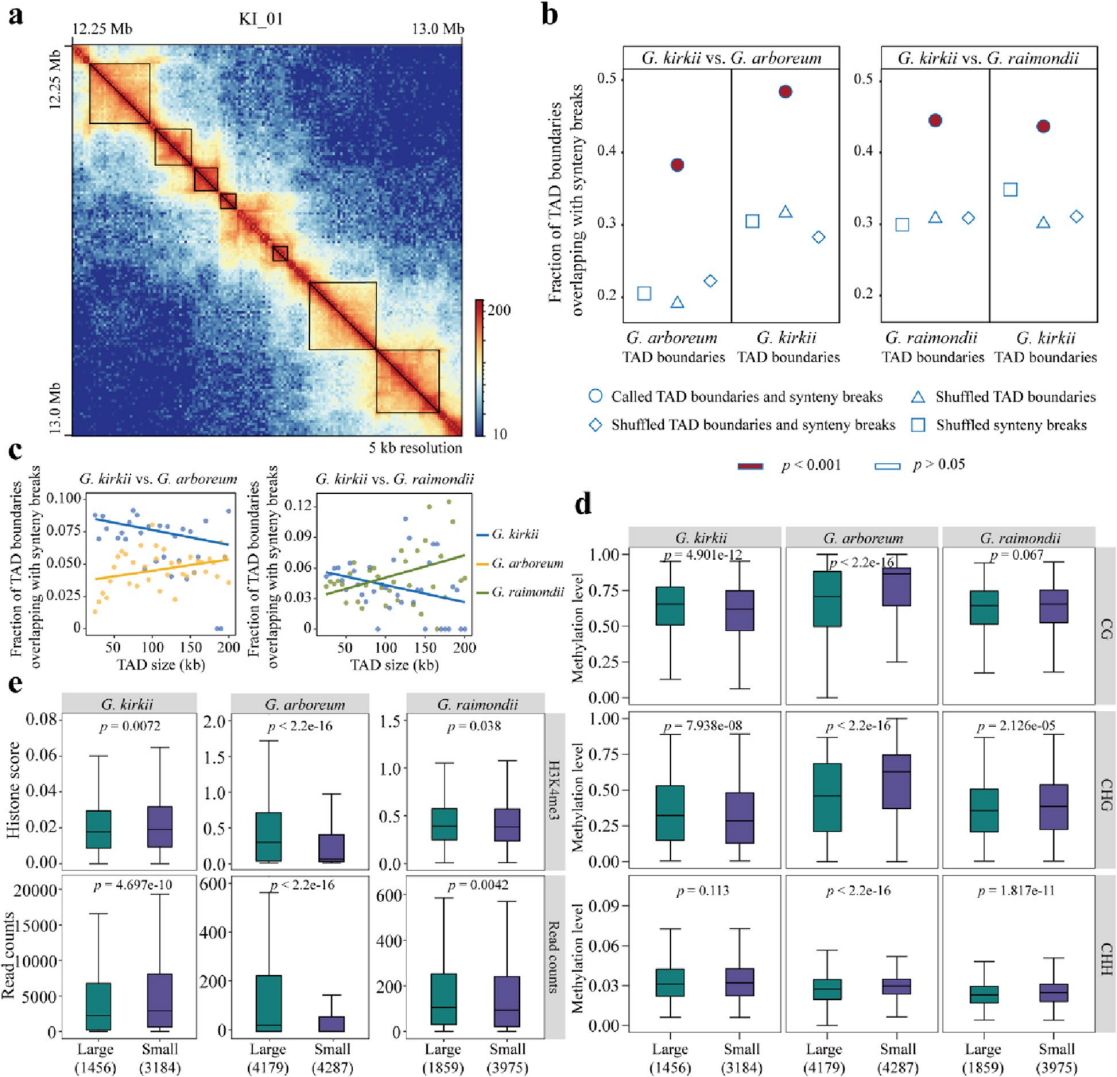
Co-localization of TAD boundaries and synteny breaks.** a** Representative chromatin interaction map involving a genomic region (750 kb) in KI_01 of *G. kirkii*, in which the component TADs are outlined by diagonal rectangles. **b** Fractions of TAD boundaries overlapping with synteny breaks identified in comparisons of *G. kirkii vs. G. arboreum* and *G. kirkii vs. G. raimondii* are statistically higher than those in multiple randomization controls, which involve groups of shuffled TAD boundaries (identified synteny breaks are maintained), shuffled synteny breaks (identified TAD boundaries are maintained), and both TAD boundaries and synteny breaks shuffled simultaneously. The maximum *p* value of Fisher’s two-tailed test in comparison to the respective control is still less than 0.001. **c** Fractions of TAD boundaries co-localizing with synteny breaks within TAD categories of different sizes in *G. kirkii*, *G. arboreum*, and *G. raimondii*, based on the regression between fraction and TAD size. **d** DNA methylation levels (in CG, CHG, and CHH contexts) in boundaries of TAD groups (large and small TADs) in *G. kirkii*, *G. arboreum*, and *G. raimondii*, respectively. **e** Abundance of active histone modification (H3K4me3) and transcribed RNA-seq reads in boundaries of TAD groups (large and small TADs) in *G. kirkii*, *G. arboreum*, and *G. raimondii*, respectively. Specific numbers of TADs in respective large and small TAD group are parenthesized at the bottom of each panel. Statistical significance was calculated using Wilcoxon’s rank sum test

Based on the Hi-C interaction matrix (at 5-kb resolution) of each individual chromosome, we characterized TAD profiles in the genomes of each species (Fig. 3a, Additional file 1: Fig. S10a). In general, we found that the number of TADs increased with genome size (*G. kirkii*: 4640; *G. raimondii*: 5834; *G. arboreum*: 8466; Additional file 1: Fig. S10b). The sizes of TADs in *G. kirkii*, *G. raimondii*, and *G. arboreum* were 61.3 kb, 63.1 kb, and 72.8 kb on average, respectively, with more small TADs (25 kb to 75 kb) with increasing genome size (Additional file 1: Fig. S10c).

To explore the potential connection between genomic rearrangements and TADs, we quantified the fraction of TAD boundaries and interior bodies co-localizing with synteny breaks identified in each species (Fig. 3b, Additional file 1: Fig. S11a). Of note, the synteny breaks identified in *G. kirkii* vs. *G. arboreum* and *G. kirkii* vs. *G. raimondii* were significantly enriched at TAD boundaries compared to the background in each species (Fisher’s two-tailed *p*-value < 0.001; Fig. 3b). This significant enrichment was not detected in either TAD interior bodies or intervals (Additional file 1: Fig. S11a, b). Further analyses revealed similar co-localization between sub-categorized inversion- and translocation-mediated breakpoints and TAD boundaries (Additional file 1: Fig. S12). These results support the idea that synteny breaks do not occur randomly relative to chromatin TADs.

Since not all TAD boundaries are associated with genomic rearrangements, the question arises as to possible mechanisms that underpin the preferential presence of TAD-boundary regions synteny breaks. To explore this, we investigated the genomic and epigenetic characteristics of TADs that have boundaries overlapping with synteny breaks (synteny break-associated TADs) versus those that do not. Initially, after binning TADs in terms of size in each species, we explored the correlation between TAD size and proportion of synteny break-associated TADs within TAD groups (Fig. 3c). Notably, a negative correlation was detected in *G. kirkii*, while positive correlations were observed for both *Gossypium* species (Fig. 3c). This species-specificity suggests that multiple factors may be involved in determining the specificity of synteny break-associated TADs. Then, according to the average TAD size of each sample, we further categorized synteny break-associated TADs into large and small TAD groups with 60 kb, 65 kb, and 75 kb in *G. kirkii*,* G. raimondii*, and *G. arboreum* as the threshold, respectively and investigated the epigenetic status of synteny break-associated TAD boundaries. We found that relative to the boundaries of large TADs, DNA methylation in three contexts (CG, CHG, and CHH) exhibited consistent hypomethylation and hypermethylation in the boundaries of small TADs in *Gossypioides* and the two *Gossypium* species, respectively (Fig. 3d). Of note, the DNA methylation levels at TAD boundaries of TAD groups exhibit contradictory trends in large and small TADs in *Gossypioides* and *Gossypium.* Considering the importance of DNA methylation in silencing transposable elements (TEs), we characterized and compared the TE abundance of large and small TAD boundaries. As expected, we found that relative to respective large TADs, the boundaries of those small TADs were significantly enriched with transposable elements (TEs) in both *G. arboreum and G. raimondii*; however, it was totally reversed in *G. kirkii*, in which the boundaries of large TAD harbor more TEs than those of small TADs (Additional file 1: Fig. S13). Moreover, active H3K4me3 histone modification and transcribed RNA abundance in the boundaries of small TADs were also higher and lower, respectively, than in large TADs in *Gossypioides* and *Gossypium* (Fig. 3e). These findings suggest that boundaries of certain TADs (small and large in *Gossypioides* and *Gossypium*, respectively) harboring open-chromatin epigenetic and active transcriptional features were associated with genetic rearrangements. The significant differences of these features in synteny break-associated TADs vs. background TADs (Additional file 1: Fig. S14) provides additional support for the preferential selection of synteny breaks in fragile TAD boundaries as well.

### TAD stability, genomic arrangements, and orthologous expression differences between *Gossypioides* and *Gossypium*

Genomic rearrangements have been shown to increase expression divergence between orthologous genes [8, 52]. In contrast, TAD stabilization might possibly play a stabilizing role with respect to expression levels of syntenic gene orthologs among closely and even distantly related animal species [6, 25, 27]. Neither the combined nor individual effects of TAD stability and synteny breaks on expression divergence of gene orthologs have been explored in plants. Accordingly, we explored these relationships in *Gossypioides* and *Gossypium*.

**Fig. 4:**
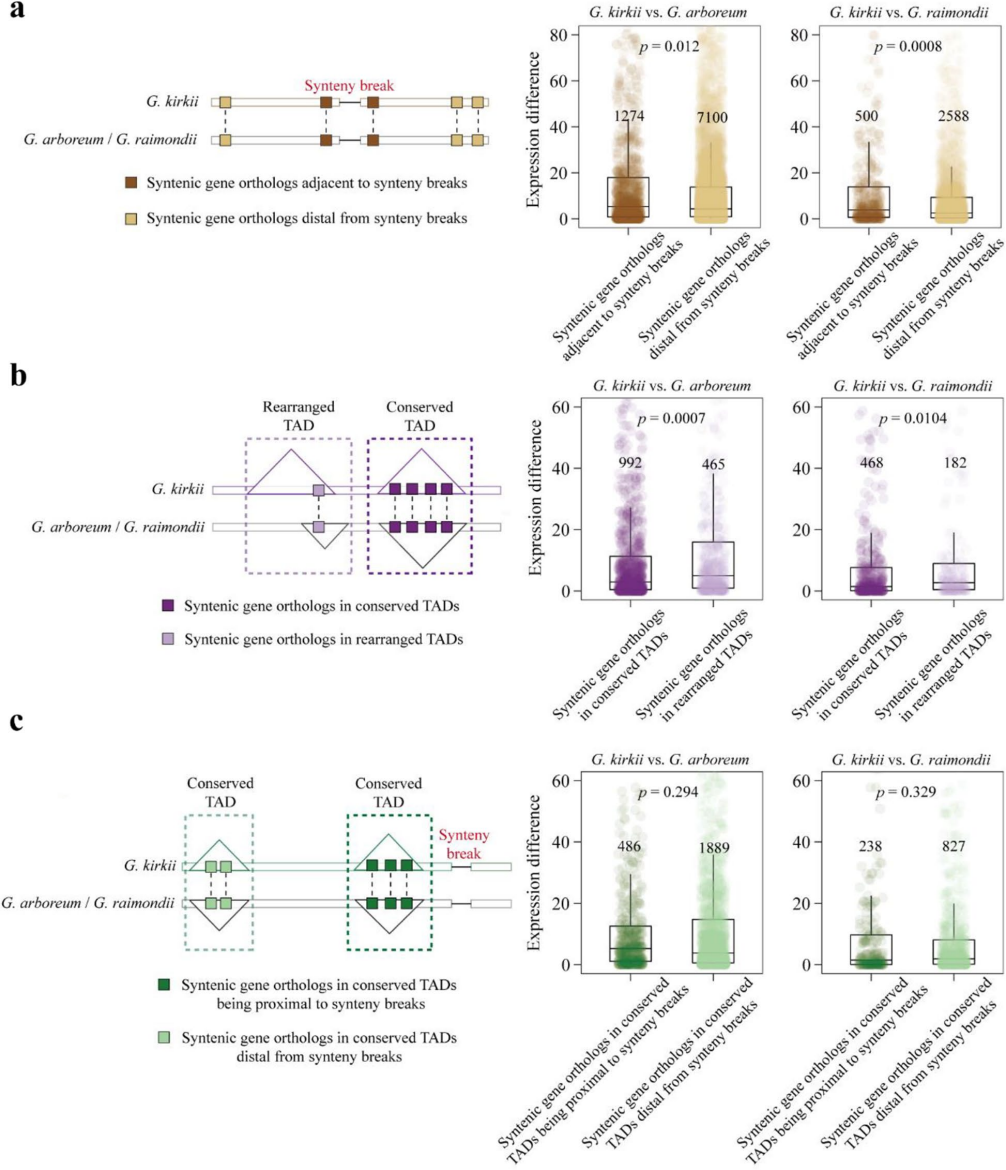
Antagonistic impact of TAD stabilization and genomic arrangements on orthologous expression divergence between *Gossypioides kirkii* and *Gossypium* species (*G. arboreum* and *G. raimondii*). **a** Expression difference between syntenic gene orthologs adjacent to synteny breaks *vs.* those distal from synteny breaks. **b** Expression difference between syntenic gene orthologs in conserved TADs *vs.* those in rearranged TADs. **c** Expression differences of syntenic gene orthologs in conserved TADs being proximal to synteny breaks *vs.* those being distal from synteny breaks. All statistical significance *p* values were calculated using Wilcoxon’s rank sum test

First, we characterized and compared expression divergence between syntenic gene orthologs adjacent to vs. distal from synteny breaks (Fig. 4a). As anticipated, syntenic gene orthologs adjacent to synteny breaks showed substantially greater expression divergence in pairwise comparisons relative to those distal from synteny breaks (Fig. 4a). To explore the impact of TAD stabilization on gene expression, we utilized a stringent definition to categorize TADs into evolutionary rearranged and conserved TADs (Methods). In total, we identified 540, 597, and 740 conserved TADs in *G. kirkii* vs. *G. arboreum*, *G. kirkii* vs. *G. raimondii*, and *G. arboreum* vs. *G. raimondii*, respectively (Additional file 1: Fig. S15a). The number of conserved TADs between paired species was associated with their phylogenetic distance, this number decreasing with an increase of divergence time (~ 5 MYA between *G. arboreum* vs. *G. raimondii* and ~ 10 MYA between *Gossypioides* and *Gossypium*; Additional file 1: Fig. S15b). Relative to syntenic gene orthologs in rearranged TADs, syntenic gene orthologs in conserved TADs exhibited lower expression divergence in both inter-generic comparisons (Fig. 4b). Collectively, these results confirm that genomic rearrangements and TAD stabilization act antagonistically in increasing and attenuating orthologous expression divergence, respectively.

To further dissect the net impact of genetic rearrangements and TAD stabilization on expression divergence of orthologs, we categorized conserved TADs into two categories, proximal and distal, in terms of their locations relative to synteny breaks. Interestingly, we found that orthologous expression divergence and their compositional epigenetic modifications (DNA methylation and active/silencing histone modifications) of syntenic genes in these two groups of TADs were not statistically different (Fig. 4c, Additional file 1: Fig. S15c). This result suggests that TAD stabilization has a greater influence than rearrangements on regulating orthologous expression divergence.

### Rearranged chromosome segments maintain ancestral *in-cis* interaction patterns

High-resolution Hi-C data can reveal *in-cis* (intra-chromosomal) point (bin)-to-point (bin) chromatin interactions [53–59]. We explored this aspect of fine-scale chromatin interaction in the context of genomic rearrangements that have arisen in our study system. Specifically, we analyzed whether the inter-chromosomal translocation and intra-chromosomal inversion in *G. arboreum* and the chromosomal fusions in *G. kirkii* established novel *in-cis* interactions in the new chromosomal contexts. With respect to *G. arboreum*, the translocation and fragment inversion are unique to this species [35], allowing both polarization of any chromatin changes as well as offering a temporal perspective on these young rearrangements (later than 0.7 MYA) [31]. Similarly, the dysploid reduction to 2n = 24 *Gossypioides* (and its sister genus *Kokia*) arose after its separation from but prior to speciation within *Gossypium* [60].

**Fig. 5:**
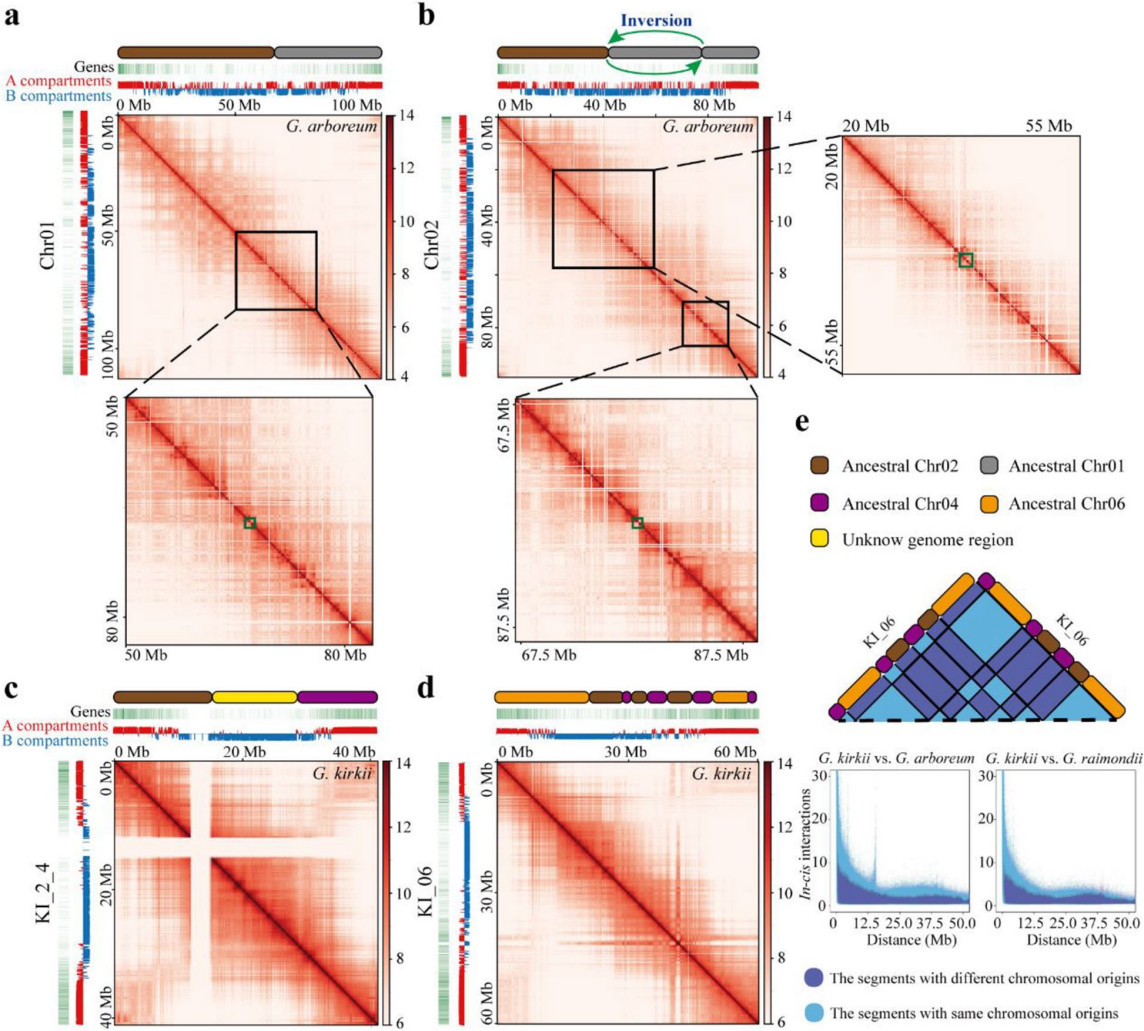
Fine-scale chromatin interaction variations associated with genomic rearrangements in *G. kirkii vs. G. arboreum* and *G. kirkii vs. G raimondii*.** a** Inter-chromosomal translocated fragments between ancestral Chr01 and Chr02 constitute the current Chr01 in *G. arboreum*. Beneath the mosaic fragments of ancestral Chr01 and Chr02 (in gray and brown, respectively) and on the left side of overall interaction map, the A/B compartment status and compositional gene density for Chr01 in *G. arboreum* are denoted. Within the zoomed-in diagonal interaction map beneath the overall interaction map, the green box denotes the disrupted diagonal interaction breakpoint corresponding to a synteny break. **b** Additional chromosomal inversion within the translocated ancestral Chr01 fragment is denoted. This introduced another synteny break into the current Chr02 in *G. arboreum*, denoted by the green box encompassing the disrupted diagonal interaction breakpoint. **c** The fused chromosomal fragments integrating ancestral Chr02 (brown), Chr04 (purple), and the unknown chromosomal fragments (yellow) in KI_2_4 of *G. kirkii*. **d** The fused chromosomal fragments integrating ancestral Chr02 (brown), Chr04 (purple), and Chr06 (orange) in KI_06 of *G. kirkii*. **e** In chromosome KI_06, the *in-cis* interactions between mosaic segments are illustrated in the upper diagram. The *in-cis* interactions (*y*-axis represents the intrachromosomal interactions that have been normalized using ICE and HiCNorm strategies) between chromosomal bins of the same chromosomal origins (as revealed in comparisons of *G. kirkii vs. G. arboreum* and *G. kirkii vs. G. raimondii*), summarized in terms of their bin-to-bin distance (*x*-axis), were statistically stronger than those between the segments of different chromosomal origins

For the relatively young translocation between Chr01 and Chr02 in *G. arboreum* (Additional file 1: Fig. S2c), the translocated chromosomal fragments maintained ancestral intra-fragmental interactions in their new chromosomal context, as evidenced by the disrupted diagonal interaction map at synteny break (Fig. 5a). Similarly, for the inverted fragment within the translocated Chr01 fragment of Chr02, it displayed frequent intra-fragmental interaction and rare interaction with the remaining Chr01 fragment (Fig. 5b).

As for the relatively old fused mosaic chromosomal fragments in *G. kirkii* (chr. KI_2_4 and chr. KI_06), their interactions with those of different chromosomal origins were observed, as reflected in the absence of demarcated interaction breakpoints close to respective synteny breaks (Fig. 5c, d). Finally, we note that *in-cis* interaction values were greater between segments with the same chromosomal origins (i.e., those from Chr02, Chr04, or Chr06 in *Gossypium*) than those having different chromosomal origins (i.e., between segments derived from Chr02 and Chr04) (Fig. 5e).

## Discussion

Genomic rearrangements occurring during evolution may have functional and hence evolutionary consequences arising from 3D chromatin architectural changes. Recent studies in animal species have explored the impacts of rearrangements on 3D chromatin architecture accompanying chromosome evolution [5–8, 26–28, 61]. These evolutionary dynamics are underexplored in plants, with little understanding of the higher-order outcomes of rearrangements with respect to hierarchical chromatin architectural features and gene expression. In this study, by integrating both chromatin interaction Hi-C and epigenomic data in two *Gossypium* species and their closely related species *Gossypioides kirkii*, we systematically characterized the evolutionary and functional connections between genomic rearrangements (represented by synteny breaks) and the alterations of hierarchical chromatin architectures.

### Rearrangements have limited effects on chromosomal territories

A key observation of the present study is that chromosomal territories were largely stable following the divergence of the three species studied here, which encompass ~ 10 million years of evolutionary divergence, and in spite of remarkable changes in genome size, structure, TE content, and gene content [31, 32] (Additional file 1: Fig. S5). Similar findings were observed based on Hi-C interaction maps in another *Gossypium* species (*G. rotundifolium*) [38] and in other diploid plant lineages that do not vary in chromosome number, such as in *Brassica* (*Brassica rapa* and *B. oleracea*) [62] and *Glycine* and *Phaseolus*. In polyploid plants, including both natural allopolyploid (*G. hirsutum*) and synthesized autopolyploids (4 × *Arabidopsis thaliana*) with altered numbers, sub-genomic chromatin interaction patterns also were similar to those of their orthologous chromosomes in diploids [39, 63]. Our findings in a plant lineage with significant genomic remodeling gives more support to a general conclusion that genomic rearrangements exert limited impacts on the overall 3D chromatin conformation at the chromosome level, which we therefore infer is under strong evolutionary constraint in different plant lineages.

### Non-random occurrence of rearrangements in open chromatin

Similar to previous results in animal species [5, 6, 26, 27], in the plant species we studied genomic re-arrangements were non-randomly distributed relative to higher-order chromatin topological compartments and local TADs (Figs. 2a and 3b). Although earlier studies reported the absence of synteny breaks in TAD bodies in other plants [61], we confirmed the absence of synteny breaks in regulatory TAD interior or body regions and their preferred location at TAD boundaries (Additional file 1: Fig. S11a). Similar findings were recently reported in pepper and their relatives [7]. These indications of conserved evolutionary constraint in both animal and plant models highlight the presumed role of TADs as functional entities that are largely maintained during chromosome evolution.

The overrepresentation of rearrangements in active, open-chromatin A compartments and at the boundaries of TADs implies that the epigenetic landscape plays a central role in facilitating or constraining genomic rearrangements (Figs. 2a and 3b, d, e, Additional file 1: Fig. S14). Several possible scenarios could explain how epigenetic modifications promote chromosomal breaks and associated rearrangements, including (*i*) relatively open chromatin regions might facilitate genomic rearrangements by increasing local chromatin fragility [28, 48, 64]; (*ii*) uncharacterized epigenetic-sensitive protein factors binding to chromatin might be involved in genomic rearrangements; and (*iii*) chromatin regions with common epigenetic states could preferentially interact, facilitating rearrangements. Insights into the relevance of these speculations will likely require both a new understanding of the molecular biology of epigenetics and recombination, along with an additional study of a diversity of species that encompass a wide range of genomic features and rearrangements.

### Rearrangements impact gene expression in the context of chromatin regulatory topology

We characterized the impact of rearrangements on the expression of adjacent syntenic genes. For syntenic genes flanking rearrangement sites, a compartment switch (B-to-A switch) (Fig. 2b, c) may reflect the potential reshaping effects of genomic rearrangements on the eu-/hetero-chromatic chromatin status. Similar prominent compartment switches to those reported here were detected in response to a genomic inversion in the sex chromosome of *Drosophila* (Y chromosome) and Ursidae (polar bear X chromosome) and autosomes of Canidae (red fox) [8, 29]. Although rearrangements for the most part do not disrupt adjacent syntenic genes, the transcriptional states of those genes may be still altered via epigenomic modifications [65–73].

These relationships between rearrangements and epigenomic/transcriptional state were clearly evident in our data (Fig. 4). Perhaps most notable is the observation, reported here, that gene expression divergence caused or facilitated by genomic rearrangements conditionally depends on proximity of orthologs to conserved TADs. In particular, we found that syntenic gene orthologs proximal to synteny breaks in conserved TADs exhibited less expression divergence than did those that were more distal from synteny breaks (Fig. 4c). It is possible that evolution has acted both on genes and their epigenomic context to ensure that they are “packaged” into insulated TADs that are protected from chromosomal breakage and their associated deleterious transcriptional effects [5, 6, 28, 49, 74–77].

### Accommodating rearranged chromosomal segments in new chromatin contexts

An important result of this study is that rearranged genomic fragments largely maintained their ancestral *in-cis* interactions. This is exemplified by the relatively young translocation between Chr01 and Chr02 in *G. arboreum* (Additional file 1: Fig. S2c). Similar maintenance of overall ancestral interaction after chromosomal rearrangement was detected in animal species as well, including *Anopheles* mosquitoes [28], muntjac deer [8], and red fox [29]. In a modern wheat cultivar (AK58) that integrated an alien chromosome segment from rye, *in-cis* intra-fragmental interaction was also conserved, with rare interaction with other wheat chromosomes [78]. These observations suggest that the maintenance of original chromatin interactions is a common feature of rearranged chromosomal fragments, across kingdoms, at least for evolutionarily relatively young rearrangements.

In addition, and in contrast to the foregoing relatively recent rearrangements, we showed novel *in-cis* interactions became established between relatively old fused segments having different chromosomal origins in *G. kirkii* (Fig. 5c, d). Similar interaction patterns were identified in old chromosomal rearrangements of the deer species *Muntiacus crinifrons* and *M. reevesi* as well [8]. The existence and importance of novel *in-cis* interaction is also supported by the absence of disrupted diagonal interaction breakpoints demarcating rearranged fragments in diploidized paleopolyploid plants, which were evolutionarily generated by multiple rounds of polyploidy and subsequent genomic fractionation [79–81]. These case studies demonstrate a temporal gradient in higher-order chromatin interactions within rearranged chromosomal fragments over the time-scales studied. In particular, young rearranged fragments largely maintain ancestral *in-cis* interaction patterns whereas over longer evolutionary time periods novel interactions become established. It will be interesting to evaluate these same relationships in other taxa and over a larger diversity of divergence times.

## Conclusions

In conclusion, we generated the first high-resolution Hi-C interaction map of *Gossypioides kirkii*, an important relative of the cotton genus. Together with our improved Hi-C interactions maps in representative *Gossypium* species, we characterized the epigenomic and expression correlates of non-random chromosomal rearrangements and 3D architectures during plant evolution. We report a remarkable enrichment of rearranged chromosomal breakpoints in euchromatic A-compartments and their co-localization with TAD boundaries. We speculate that genomic re-arrangements could affect gene expression, which is antagonized by TAD stabilization. Finally, we illustrate an evolutionary temporal dependence to these phenomena. Overall, our study expands our current understanding of the interplay between chromosome evolution and hierarchical 3D chromatin architecture, with implications for highly shuffled genomes across the grand sweep of evolutionary divergence in plants.

## Methods

### Plant materials

Plants of *Gossypioides kirkii*, *Gossypium arboreum* (A_2_), and *Gossypium raimondii* (D_5_) were cultivated in the greenhouse at Northeast Normal University in Changchun, China. Fresh young leaves were collected individually and immediately frozen in liquid nitrogen. Leaves were harvested, mixed, and divided into different replicates, which were input into Hi-C (high-throughput chromosome conformation capture), WGBS-seq (Whole genome bisulfite sequencing), RNA-seq (RNA sequencing), and ChIP-seq (chromatin immunoprecipitation followed by sequencing) experiments, respectively.

### Hi-C experiment, library construction, and sequencing

Following a protocol described previously, with modifications (Belton et al., 2012), we constructed Hi-C libraries using leaves as inputs. Fresh leaves of each sample were chopped with sharp blades and fixed with 2% formaldehyde solution for 15 min at room temperature, frozen in liquid nitrogen, and used for a Hi-C pipeline described in Dong et al. [82], using the four-cutter restriction enzyme *Mbo*I at 37 °C on a rocking platform. Hi-C libraries were then sequenced on an Illumina HiSeq X Ten with 150 bp paired reads.

### Construction and comparison of Hi-C interaction maps

Raw Hi-C reads in FASTQ files were processed to remove low-quality reads and trim adapters, and clean sequencing reads were aligned to the *G. arboreum* genome (https://www.cottongen.org/species/Gossypium_arboreum/CRI-A2_genome_v1.0), *G. raimondii* genome (https://www.cottongen.org/species/Gossypium_raimondii/NSF-D5), and *G. kirkii* genome (https://www.cottongen.org/species/Gossypioides_kirkii/ISU_kirkii) by bowtie2 with default settings [83]. Uniquely mapped reads were assigned to respective restriction fragments [84]. Ratios of theoretically digested genomic fragments supported by PE Hi-C reads were estimated. The Hi-C interaction matrix was constructed following methods described in a previous study [11, 82]. The ICE (Iterative Correction and Eigenvector) method was used to correct the Hi-C bias caused by restriction fragment length, GC content, and the mapping ability of sequenced reads [85]. We also used the improved HiCNorm method (based on the Poisson regression) to remove biases introduced by the number of mapped sequences flanking all restriction enzyme cutting sites (HiCNorm: removing biases). Hi-C interaction matrices enclosing paired equal-sized bins at various resolutions were calculated using Hi-C-Pro (in the default settings) [84]. Pearson correlations between Hi-C interaction maps in different replicates were calculated to verify reproducibility. Because correlation was significant for each sample, valid Hi-C data of different replicates were combined, which were input for the construction of Hi-C interaction matrices. The HiC-Pro contact matrices in different resolutions were transformed into different formats for specific analysis using HiCExplorer (https://github.com/deeptools/HiCExplorer/) by hicTransform. Finally, genome-wide Hi-C interaction matrices were visualized by hicPlotMatrix and the resolution of Hi-C interaction maps was evaluated as previously outlined [86]. The resolution of Hi-C data sets was estimated as 5 kb for *G. arboreum*, 1 kb for *G. raimondii*, and 1 kb for *G. kirkii*.

The enrichment of *trans*-interactions between a pair of chromosomes was calculated as described in an earlier study [87]. In short, the values are given as the log_2_ ratio of the observed to the expected value. Moreover, we utilized an in-house Perl script to extract the *in-cis* interactions between chromosomal bins of the same and different chromosomal origins in Fig. 5e from the contact matrix, which has been normalized using the ICE (Iterative Correction and Eigenvector) and HiCNorm (based on the Poisson regression) strategies.

### Identification of genomic compartments and TADs

Compartment analysis was performed at the 50-kb resolution as previously described [10]. PCA was applied to predicted A and B compartments on the corrected matrix of each chromosome in each species by using the matrix2 compartment module in cworld software (https://github.com/dekkerlab/cworld-dekker). The eigenvalues of the first principle component (PC1) along each chromosome were utilized to parse the bins into two types of compartments. Chromosomal bins with a positive or negative first eigenvector denoted compartments A and B.

TADs (*T*opologically *A*ssociated *D*omains) were identified on 5-kb corrected Hi-C maps in each species by adopting the method proposed by Liu et al. [51]. Based on such identification method, TAD boundaries correspond to the regions flanking (± 10 kb) to the respective bottom point of the TAD interaction triangle; TAD bodies are those interior regions enclosed by two TAD boundaries; TAD intervals are those regions located between two TADs. If the ratio of overlapping syntenic genes to the total number of syntenic genes in the compared region exceeded 50%, the region was considered a conserved TAD, and only domains containing more than four syntenic genes were retained.

### Detection of synteny breaks

To detect potential synteny breaks in paired comparisons (*G. kirkii* vs. *G. arboreum* and *G. kirkii* vs. *G. raimondii*), we initially completed mutual BLASTP between whole-genomic genic proteins in paired species to identify their gene homologs (*e*-value ≤ 1e − 5; top 5 matches) [88]; second, we adopted ColinearScan to locate colinear syntenic blocks between paired species (the genomic gap between syntenic blocks enclosing > 30 non-syntenic genes and *p*-value < 0.05) [89]; third, the ends of colinear syntenic blocks enclosing a certain minimal number of syntenic genes were defined as the borders of synteny breaks. Several empirical values for gene number (5, 8, 10, 20, and 30) were set and their outputs of synteny breaks in paired comparisons were summarized in curves (Additional file 1: Fig. S1). Notably, two values (8 and 10) were close to the inflection point in the curves. To reduce false positive inferences of synteny breaks, we therefore adopted a minimum value of 10 genes for inferences of synteny breaks in our analyses.

### Overlaps between synteny breaks and TAD boundaries, interior bodies, and intervals

To investigate the association of synteny breaks with TAD boundaries, we explored whether they have positional co-localization that is statistically higher than that for randomized backgrounds. The randomized background was obtained by shuffling the position of synteny breaks and/or TAD boundaries along the genome while keeping the same region size distribution within the same chromosomes (“*bedtools shuffle -noOverlapping -chrom -g chrom.size*” with the appropriate chromosome sizes attributed to the “*-g*” parameter depending on the species). We quantified the overlap of synteny breaks with TAD boundaries and randomized background, respectively (“*bedtools intersect*”) and statistical significance was tested by Fisher exact’s test (“*bedtools fisher*” with the “*-g*” parameter for each species). We adopted a similar method to investigate the association of synteny breaks with TAD interior bodies and TAD intervals.

### RNA-seq and data analysis

Three *Gossypieae* species were grown in the greenhouse at Northeast Normal University in Changchun, China. Leaves were harvested, mixed, and divided into different replicates. For each replicate, leaves were immediately frozen in liquid nitrogen. Total RNA was extracted from three replicates using the Concert Plant RNA Reagent (Invitrogen) according to the manufacturer’s instructions. Illumina Hiseq 4000 libraries were prepared for each replicate and sequenced (PE150) at BerryGenomics (Beijing, China). FASTX-Toolkit (http://hannonlab.cshl.edu/fastx_toolkit/) software was used for data filtering and quality control. Then the reference index was established by using hisat2 software [90] and the processed data was compared to the corresponding genome with default parameters. RPKM (Reads Per Kilobase of transcript per Million fragments sequenced) for each gene were calculated by cufflinks [91] and the average of three biological replicates was taken as the expression level of each gene. In order to directly calculate the expression difference of syntenic gene orthologs, we calculated the absolute expression difference (|e_1_ − e_2_|, e_1_: expression level of syntenic gene orthologs in *G. arboreum*/*G. raimondii*; e_2_: expression level of syntenic gene orthologs in *G. kirkii*) between syntenic gene orthologs in *G. arboreum* vs. *G. kirkii* and *G. raimondii* vs. *G. kirkii* based on read counts (log_10_RPKM) within each *Gossypieae* species.

### WGBS-seq and data analysis

Total DNA was extracted from three replicates of 3 g leaves using the Qiagen Plant DNeasy Kit (Qiagen). Whole genome bisulfite sequencing (WGBS-seq) for each replicate was conducted (PE150) on the Illumina Hiseq 4000 platform at BerryGenomics (Beijing, China) with standard protocols. After filtering out adaptors and low-quality reads (keeping reads with > 80% of bases having a quality score > than 20) using FASTX-Toolkit (http://hannonlab.cshl.edu/fastx_toolkit/), those cleaned reads were mapped to each genome using Bismark [92], and the read pairs with high-quality alignments (mapping quality > 30) were kept. The potentially methylated cytosine sites were extracted with a Bismark methylation extractor. Cytosine sites with ≥ 5 mapped reads were utilized for downstream analysis.

### ChIP-seq and data analysis

Tissue fixation and nuclei extraction were performed according to previous studies [93, 94]. Nuclei from 1 g leaves were used for one round of ChIP. The ChIP experiments essentially followed Wang et al. (year) with minor changes. In brief, nuclei were pelleted by centrifugation and washed twice using isolation buffer and suspended in nuclear digestion buffer; extracted nuclei were sheared to an average size of 150 bp with MNase (Sigma N3755). The digested fragments were immunoprecipitated with 3 μg of anti-H3K4me3 (Abcam 1012) antibodies, 3 μg of anti-H3K27me3 (Abcam 6002) antibodies, and 3 μg of anti-H3K9me2 (Abcam 1220) antibodies. After overnight incubation at 4 °C, the antibodies were recovered with 25μL rProtein A Magnetic Beads (ab214286) followed by a series of washing steps. ChIP-ed DNA was released for end-repairing, A-tailing, adaptor ligation, and library amplification steps were following described previously [93, 94]. The ChIP libraries were sequenced (PE150) on the Illumina NovaSeq 6000 platform. The ChIP-seq reads were aligned to each genome using bowtie2 with default parameter setting [83]. Only read pairs with high-quality alignments (Mapping quality > 20) were retained for downstream analyses. For histone score, we calculated the log_2_ ratio of ChIP versus input as the histone signal using deeptools bamCompare [95]. TAD were delimited into the same number of genomic regions and the histone score of each genomic region was calculated using deeptools computeMatrix [95].

## Supplementary Information

**Additional file 1:** **Fig. S1.** Synteny breaks identified by setting different minimal number of syntenic genes (5, 8, 10, 20, and 30) enclosed in colinear syntenic blocks in paired comparisons (*G. kirkii vs. G. arboreum *and* G. kirkii vs. G. raimondii*). **Fig. S2. **Genomic gene synteny identified in *G. kirkii* *vs.* *G. arboreum* and *G. kirkii* *vs.* *G. raimondii* genomes. **Fig. S3.** Characterization of synteny breaks in diploid *Gossypieae* species. **Fig. S4.** Reproducibility and resolution of Hi-C data. **Fig. S5.** Genome-wide Hi-C contact maps constructed in *G. arboreum*, *G. raimondii*, and* G. kirkii*. **Fig. S6.** Relative distribution of orthologous chromosomes that were not involved in inter-chromosomal rearrangements mediating the descending dysploidy in *Gossypioides kirkii*. **Fig. S7.** The chromosomal landscape of genomic and epigenomic features within identified A/B compartments in* G. kirkii*, *G. arboreum*, and *G. raimondii*. **Fig. S8. **DNA methylation and histone modification near (±2 kb) the gene body of stable (A/B compartment status stable) and switched genes (A/B compartment status switching/transitions). **Fig. S9. **Representative IGV snapshots illustrating the A/B compartment, epigenetic features (DNA methylation and histone modifications), and gene models around the synteny break in *G. arboreum vs. G. kirkii* (top) and* G. raimondii vs. G. kirkii* (bottom). **Fig. S10.** Profile of TADs identified in *G. kirkii*, *G. arboreum*, and *G. raimondii*, respectively. **Fig. S11.** No statistically significant co-localization between synteny breaks and TADs interior bodies and intervals. **Fig. S12. **Fractions of TAD boundaries overlapping with breakpoints of inversion and translocation identified in comparisons of *G. kirkii vs. G. arboreum* and *G. kirkii vs. G. raimondii* are statistically higher than those randomization controls, which involve groups of shuffled TAD boundaries, shuffled breakpoints, and both TAD boundaries and breakpoints shuffled simultaneously. **Fig. S13.** Abundance of transposable element (TEs) in boundaries of TAD groups (large and small TADs) in *G. kirkii*, *G. arboreum*, and *G. raimondii*, respectively. **Fig. S14.** Open-chromatin epigenetic and active transcriptional features of TAD boundaries co-localizing with synteny breaks. **Fig. S15.** The relationships between genomic rearrangements and epigenetic modifications of syntenic genes for conserved TADs. **Table S1.** Summary of clean Hi-C reads. **Table S2. **Summary of uniquely mapped Hi-C reads. **Table S3. **Summary of valid Hi-C reads. **Table S4. **Pearson correlations of overall chromosomal distributions in *G. kirkii*, *G. arboreum*, and *G. raimondii*. **Table S5. **Epigenetic data from public databases.

## Data Availability

The sequencing data sets generated within the current study have been submitted into the NCBI Sequence Read Archive (SRA; http://www.ncbi.nlm.nih.gov/sra) under accession number PRJNA865242 (https://www.ncbi.nlm.nih.gov/bioproject/?term=PRJNA865242) [96]. All other public epigenetic data are tabulated in Additional file 1: Table S5 (https://trace.ncbi.nlm.nih.gov/Traces/?study=SRP114409) [39].

## Abbreviations

3D: Three-dimensional
Hi-C: High-throughput chromosome conformation capture
CT: Chromosomal territories
TAD: Topologically associated domain
MYA: Million years ago
GO: Gene ontology
PE: Paired-end
ICE: Iterative correction and eigenvector
PCA: Principal component analysis
TE: Transposable element
WGBS-seq: Whole genome bisulfite sequencing
RNA-seq: RNA sequencing
ChIP-seq: Chromatin immunoprecipitation followed by sequencing
PC1: First principle component
RPKM: Reads per kilobase of transcript per million fragments sequenced
