# Supplementary material for: Genomic rearrangements and evolutionary changes in 3D chromatin topologies in the cotton tribe (Gossypieae)
Source: BMC Biol. 2023 Mar 20;21:56. doi: 10.1186/s12915-023-01560-y (PMC10029228; doi:10.1186/s12915-023-01560-y)
Supplement: Supplementary file 1 — Additional file 1: Fig. S1. Synteny breaks identified by setting different minimal number of syntenic genes (5, 8, 10, 20, and 30) enclosed in colinear syntenic blocks in paired comparisons (G. kirkii vs. G. arboreum and G. kirkii vs. G. raimondii). Fig. S2. Genomic gene synteny identified in G. kirkii vs. G. arboreum and G. kirkii vs. G. raimondii genomes. Fig. S3. Characterization of synteny breaks in diploid Gossypieae species. Fig. S4. Reproducibility and resolution of Hi-C data. Fig. S5. Genome-wide Hi-C contact maps constructed in G. arboreum, G. raimondii, and G. kirkii. Fig. S6. Relative distribution of orthologous chromosomes that were not involved in inter-chromosomal rearrangements mediating the descending dysploidy in Gossypioides kirkii. Fig. S7. The chromosomal landscape of genomic and epigenomic features within identified A/B compartments in G. kirkii, G. arboreum, and G. raimondii. Fig. S8. DNA methylation and histone modification near (±2 kb) the gene body of stable (A/B compartment status stable) and switched genes (A/B compartment status switching/transitions). Fig. S9. Representative IGV snapshots illustrating the A/B compartment, epigenetic features (DNA methylation and histone modifications), and gene models around the synteny break in G. arboreum vs. G. kirkii (top) and G. raimondii vs. G. kirkii (bottom). Fig. S10. Profile of TADs identified in G. kirkii, G. arboreum, and G. raimondii, respectively. Fig. S11. No statistically significant co-localization between synteny breaks and TADs interior bodies and intervals. Fig. S12. Fractions of TAD boundaries overlapping with breakpoints of inversion and translocation identified in comparisons of G. kirkii vs. G. arboreum and G. kirkii vs. G. raimondii are statistically higher than those randomization controls, which involve groups of shuffled TAD boundaries, shuffled breakpoints, and both TAD boundaries and breakpoints shuffled simultaneously. Fig. S13. Abundance of transposable element (TEs) in bo [file 12915_2023_1560_MOESM1_ESM.docx]

**Supplementary information**

**
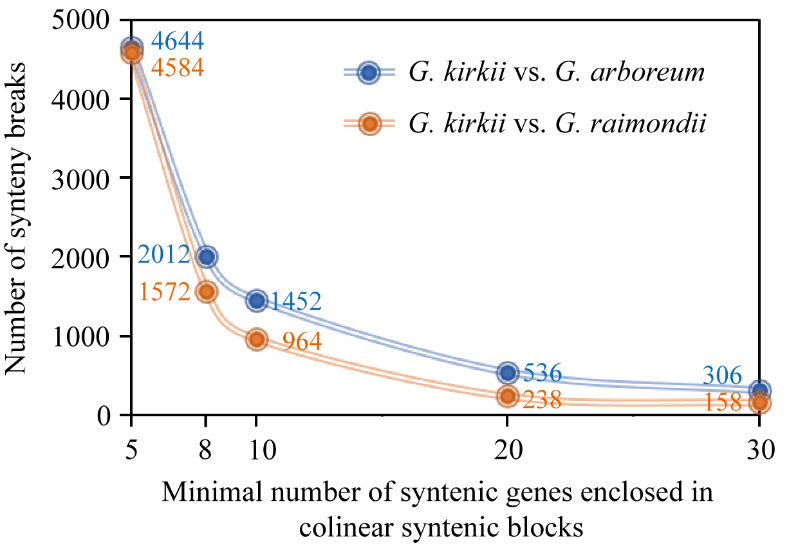
**

**Figure S1. Synteny breaks identified by setting different minimal number of syntenic genes (5, 8, 10, 20, and 30) enclosed in colinear syntenic blocks in paired comparisons (*G. kirkii vs. G. arboreum* and *G. kirkii vs. G. raimondii*).**

**
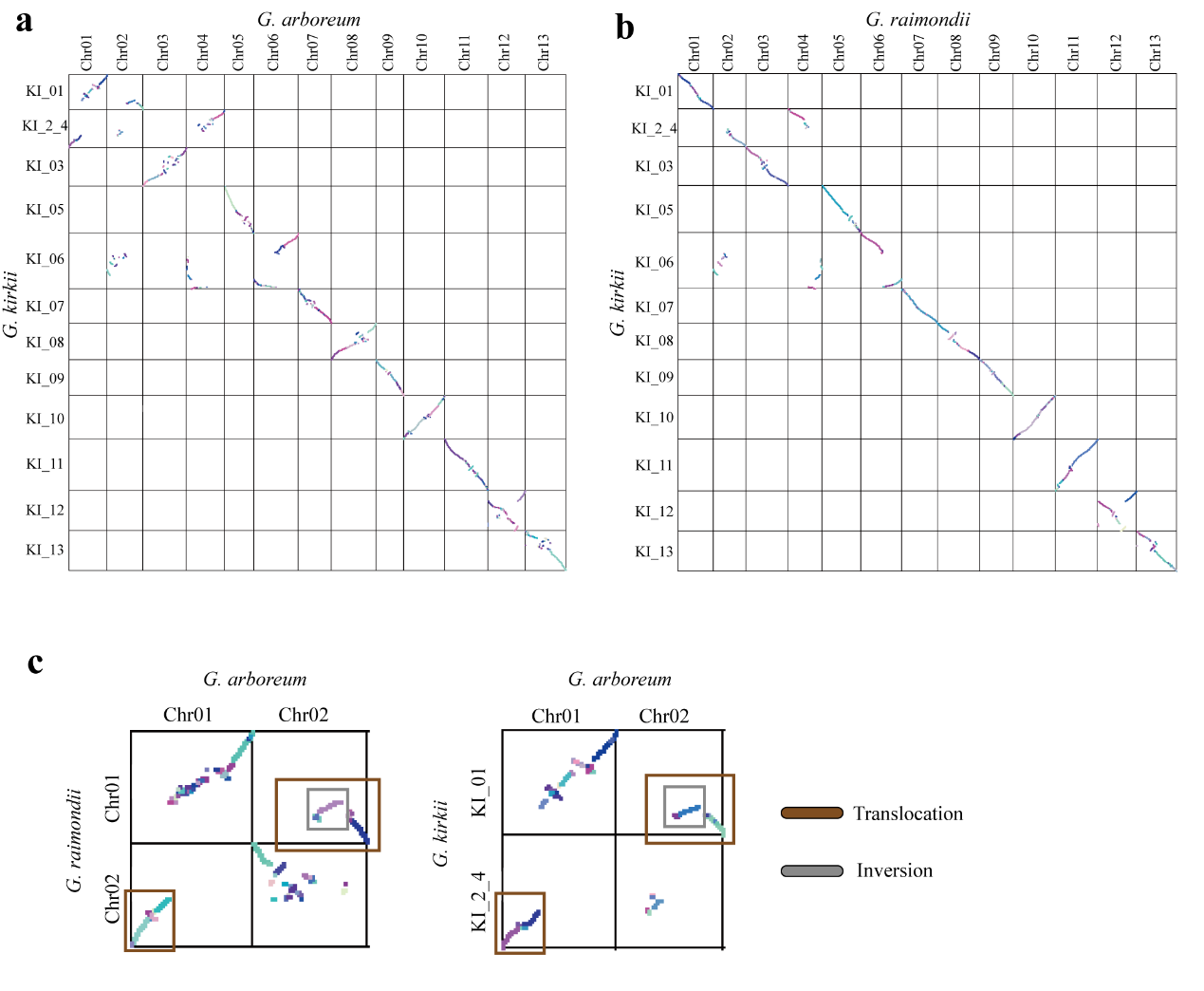
**

**Figure S2. Genomic gene synteny identified in *G. kirkii* *vs.* *G. arboreum* and *G. kirkii* *vs.* *G. raimondii* genomes. a** Dot plot illustrating whole-genomic gene synteny between *G. arboreum* (x-axis) and *G. kirkii* (y-axis). **b** Dot plot illustrating whole-genomic gene synteny between *G. raimondii* (x-axis) and *G. kirkii* (y-axis). Genomic alignment was carried out using the MCScanX package. **c** Dot plot illustrating chromosomal gene synteny between Chr01 and Chr02 in *G. arboreum* *vs.* *G. raimondii* and *G. arboreum* *vs.* *G. kirkii*. The translocation between Chr01 and Chr02 segments and inversion within Chr02 are uniquely identified in *G. arboreum* and illustrated in mosaic colored bars. Genomes were downloaded from the CottonGen website (<https://www.cottongen.org/data/download>).


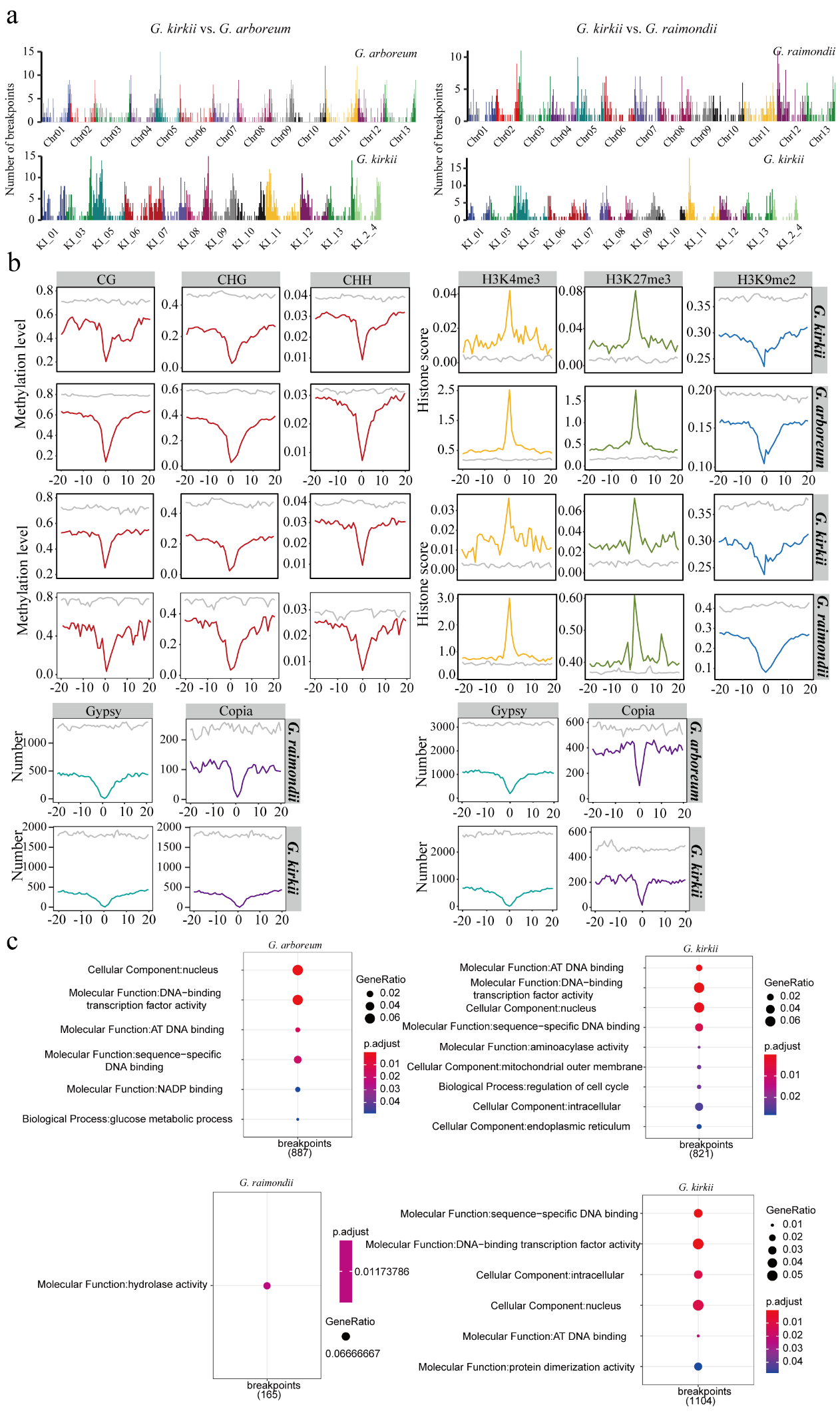


**Figure S3. Characterization of synteny breaks in diploid *Gossypieae* species.** **a** The distribution of breakpoints across respective genome. **b** Epigenetic signatures of identified synteny breaks in *G. kirkii vs. G. arboreum* and *G. kirkii vs. G. raimondii*. DNA methylation levels (in CG, CHG, and CHH contexts; red curves), histone modification scores (H3K4me3, H3K27me3, and H3K9me2; yellow, green, and blue curves), and transposable elements (Copia and Gypsy; bottom of 8 panels) number were smoothed in loess curves spanning 1-kb bins across respective synteny break (±20 kb; in colored curves) and randomized genomic regions (in grey), which illustrate the unique enrichment or depletion of these epigenetic marks. **c** GO enrichment of genes adjacent to synteny breaks in *G. arboreum*, *G. raimondii*, and *G. kirkii*.


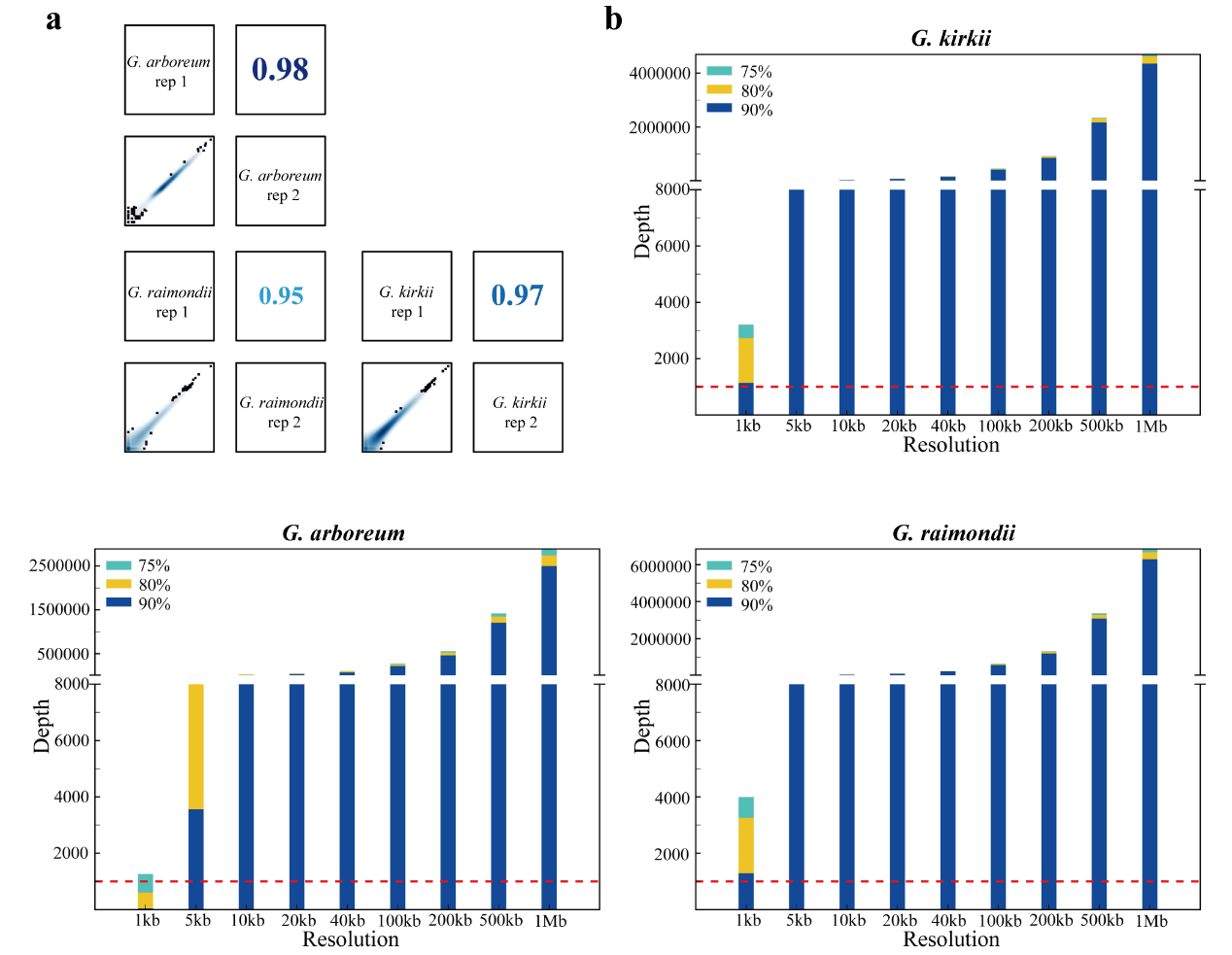


**Figure S4. Reproducibility and resolution of Hi-C data. a** Pearson correlations among Hi-C replicates of *G. kirkii*, *G. arboreum*, and *G. raimondii*. **b** Resolution of *G. kirkii*, *G. arboreum*, and *G. raimondii* Hi-C maps.


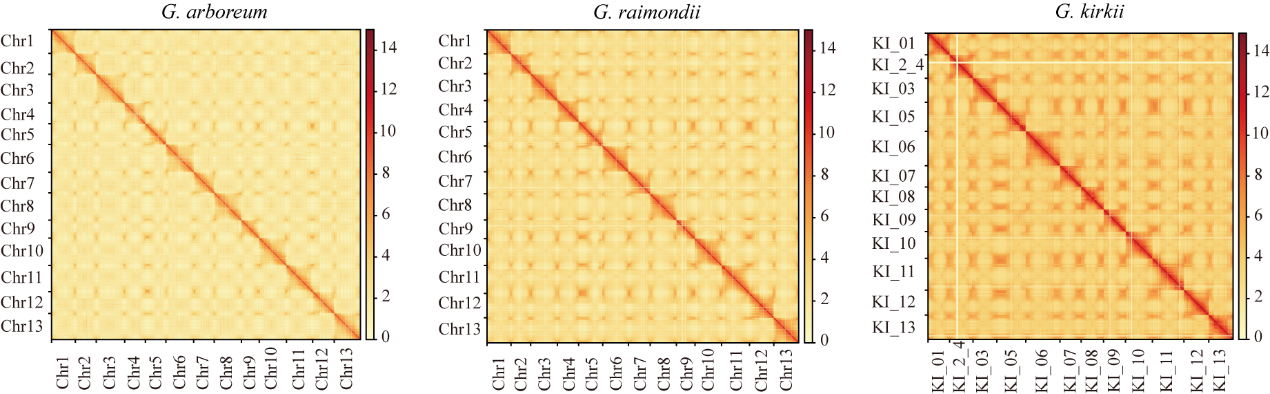


**Figure S5. Genome-wide Hi-C contact maps constructed in *G. arboreum*, *G. raimondii*, and *G. kirkii*.** Hi-C interaction matrix of *G. arboreum*, *G. raimondii*, and *G. kirkii* at 100 kb resolution are illustrated in heatmaps. In the heatmaps, chromatin bins interacting in higher contact frequency are in darker red and *vice versa*.

**
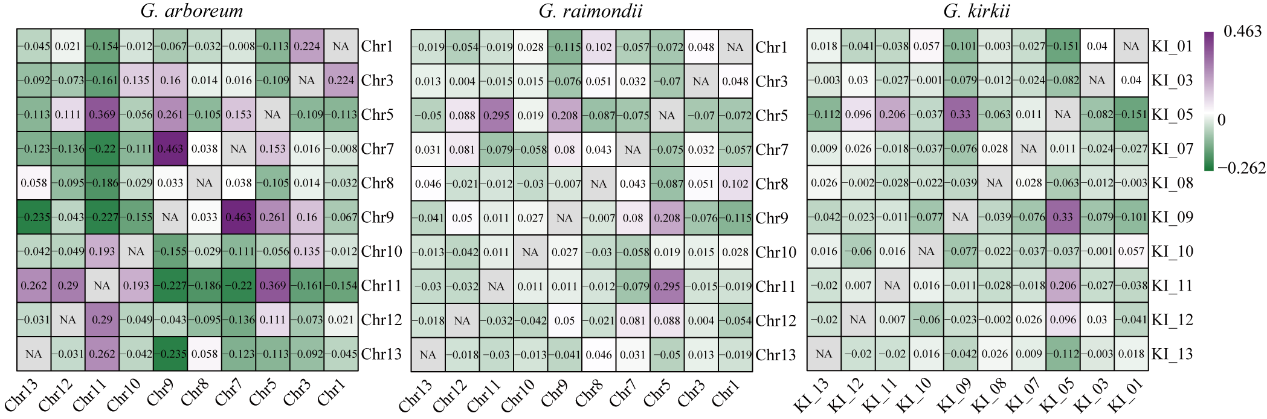
**

**Figure S6. Relative distribution of orthologous chromosomes that were not involved in inter-chromosomal rearrangements mediating the descending dysploidy in *Gossypioides kirkii*.** For each pair of chromosomes, their relative chromosome distribution (reflecting their occupied chromosome territory) was evaluated as the log_2_ transformed ratio of observed *vs.* expected pairwise inter-chromosomal interactions in *G. arboreum*, *G. raimondii*, and *G. kirkii* (Methods).

**
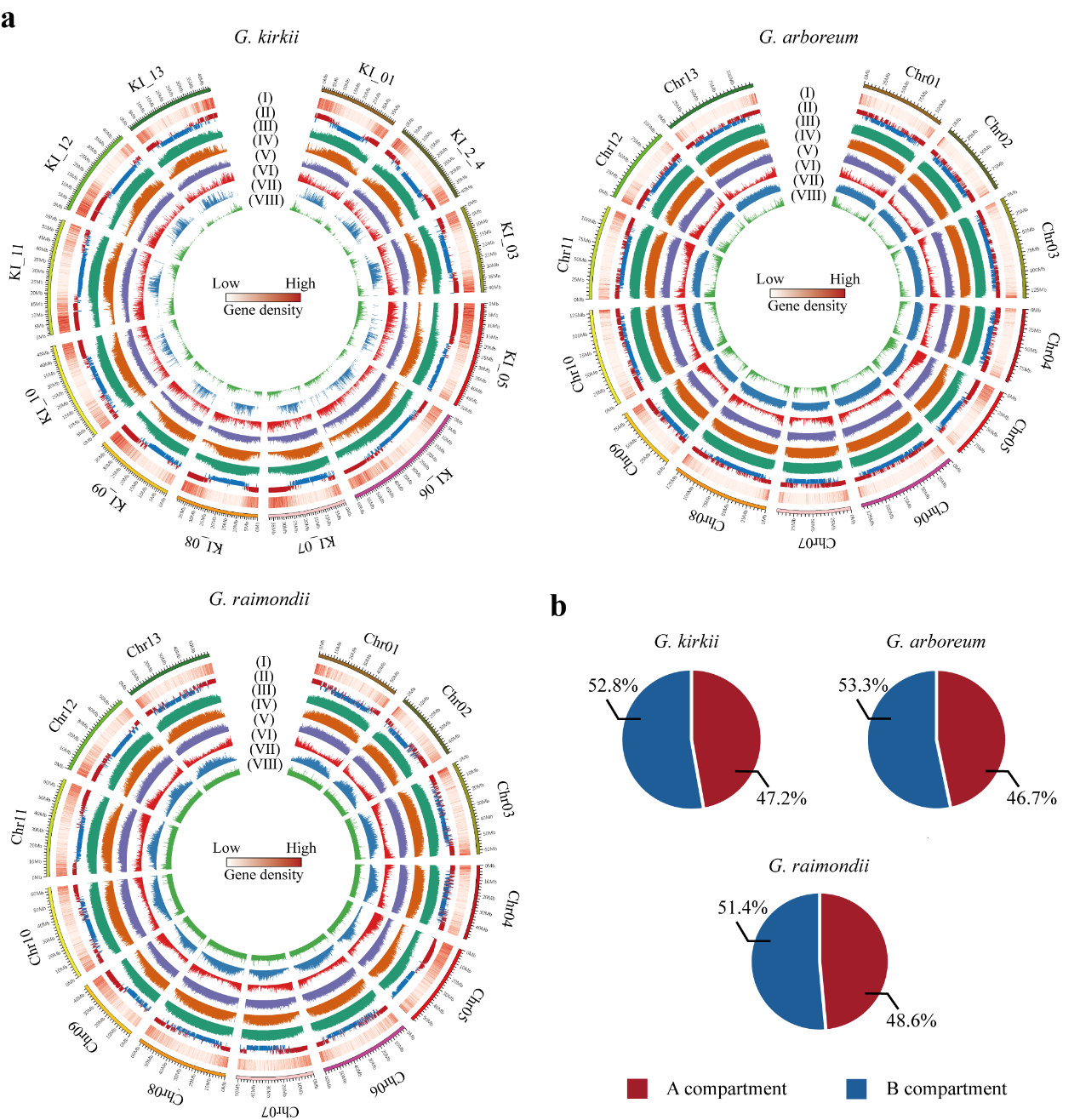
**

**Figure S7. The chromosomal landscape of genomic and epigenomic features within identified A/B compartments in** ***G. kirkii*, *G. arboreum*, and *G. raimondii*. a** Circos plot showing chromosome-level features of *G. kirkii*, *G. arboreum*, and *G. raimondii*. Rings represent the i: density of protein-coding genes (indicated by the color scale), ii: PC1 values, iii: CG methylation level, iv: CHG methylation level, v: CHH methylation level, vi: the enrichment of H3K4me3 modification, vii: the enrichment of H3K9me2 modification, viii: the enrichment of H3K27me3 modification. For the signal strength of each ring, respective signal was calculated in 50-kb sliding window. **b** Pie chart summarizing the percentages of A/B compartments identified in *G. kirkii* and two *Gossypium* species.


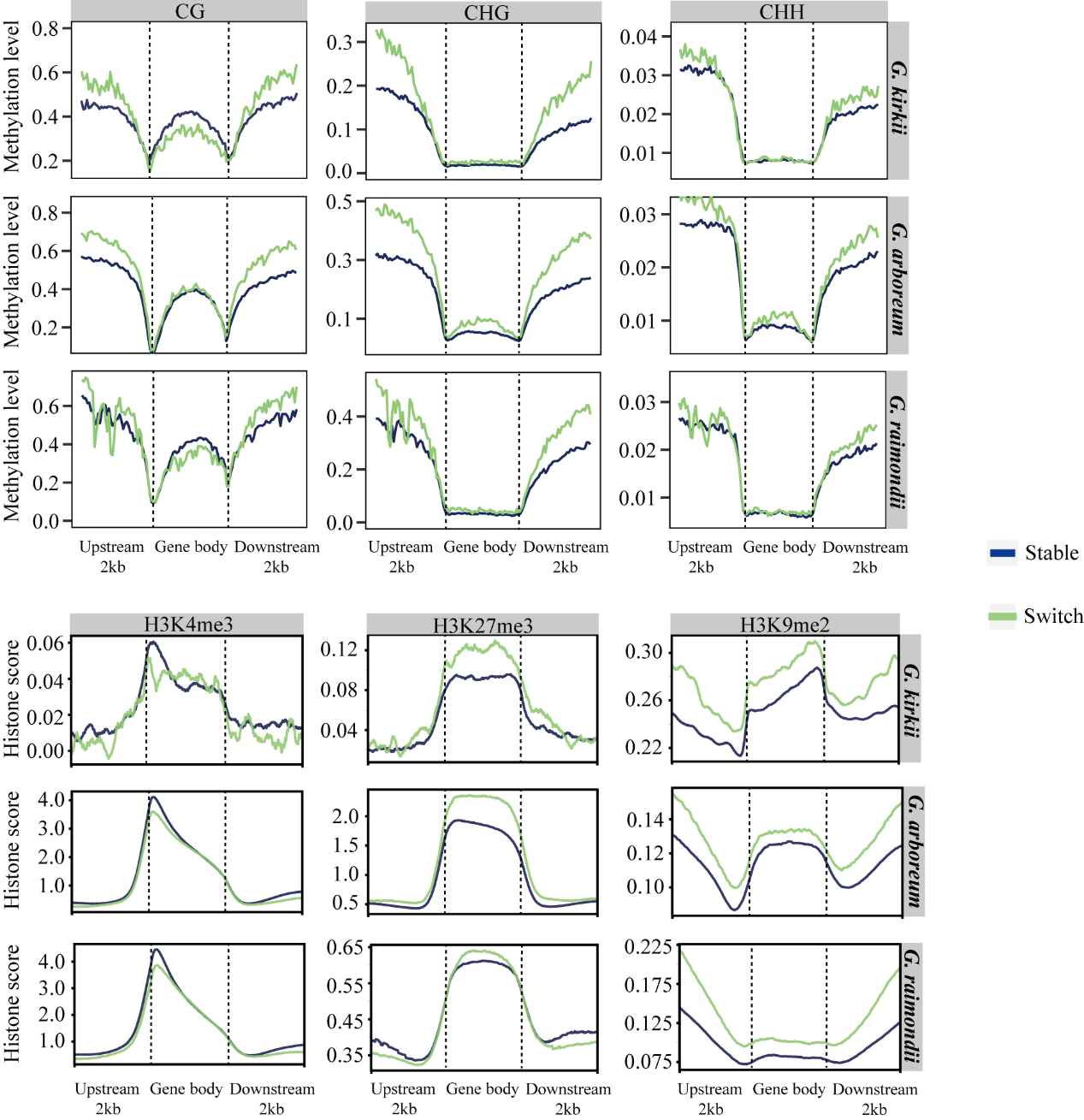


**Figure S8. DNA methylation and histone modification near (****±2 kb) the gene body of stable (A/B compartment status stable) and switched genes (A/B compartment status switching/transitions).**


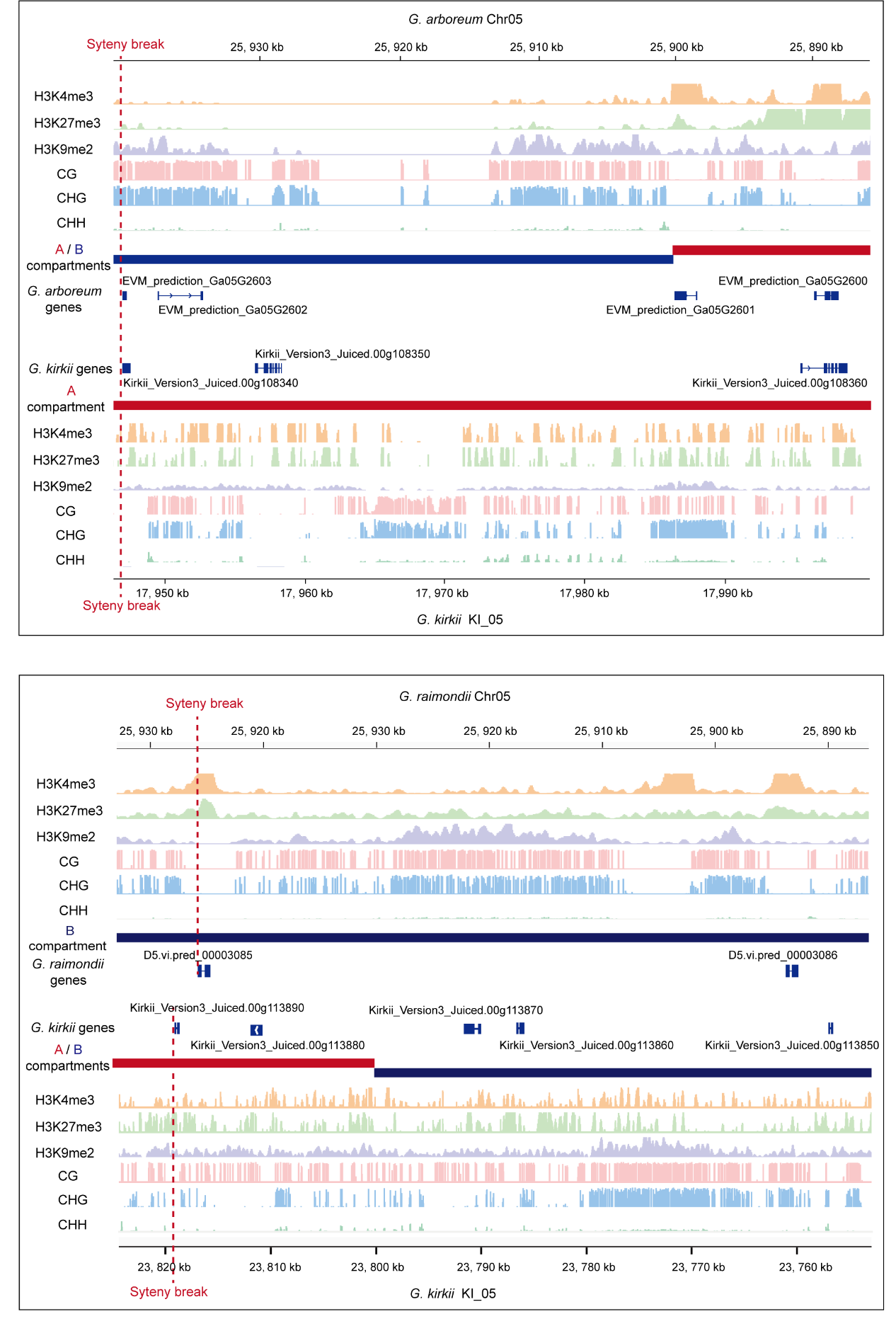


**Figure S9. Representative IGV snapshots illustrating the A/B compartment, epigenetic features (DNA methylation and histone modifications), and gene models around the synteny break in *G. arboreum vs. G. kirkii* (top) and *G. raimondii vs. G. kirkii* (bottom).**


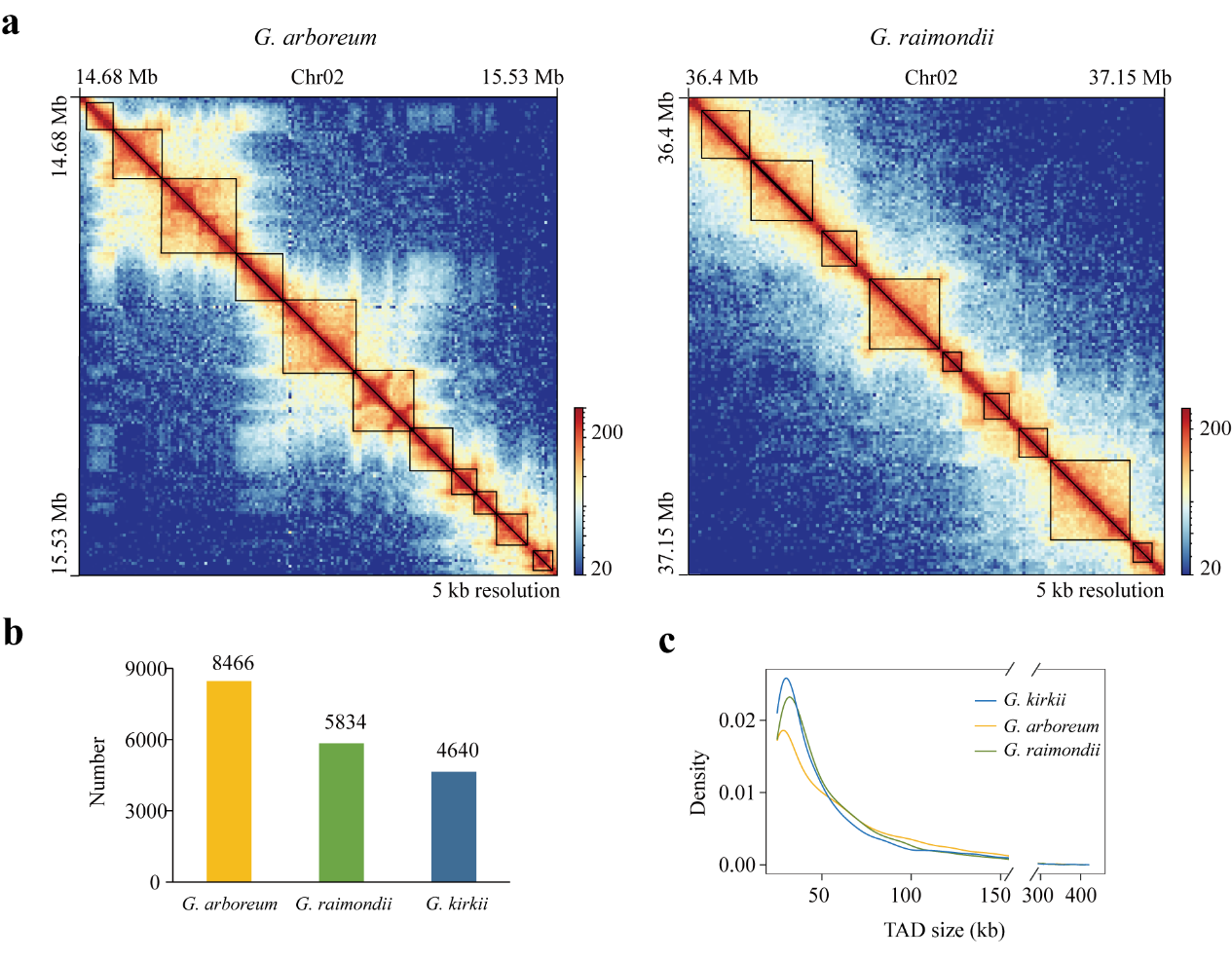


**Figure S10. Profile of TADs identified in *G. kirkii*, *G. arboreum*, and *G. raimondii*,** **respectively.** **a** Representative chromatin interaction maps involving genomic regions in Chr02 of *G. arboreum* and *G. raimondii*, in which the component TADs are outlined by diagonal rectangles. **b** Number of TAD identified in respective species. **c** Density distribution of TAD in groups of various sizes in *G. kirkii*, *G. arboreum*, and *G. raimondii*.


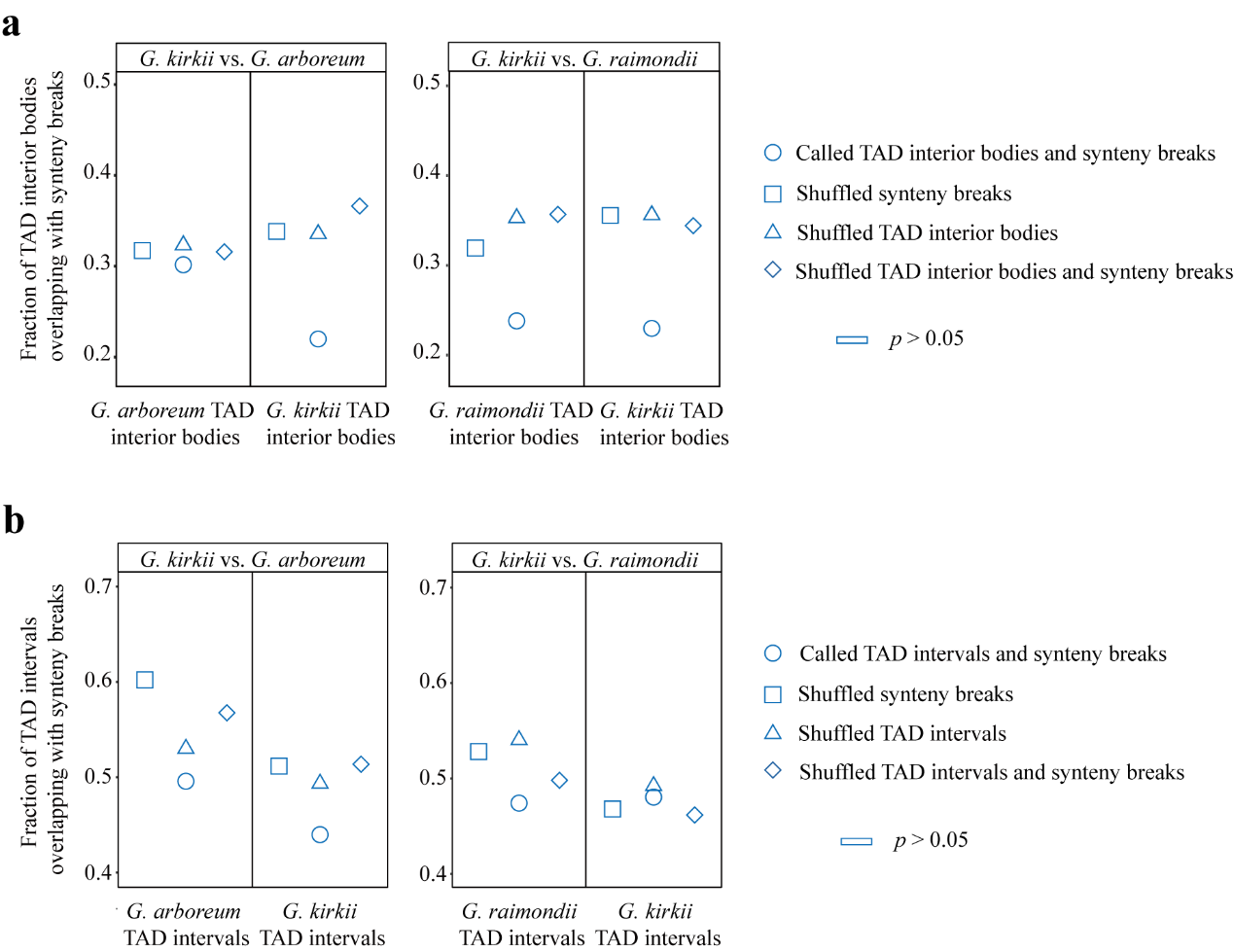


**Figure S11. No statistically significant co-localization between synteny breaks and TADs interior bodies and intervals. a** Fractions of TAD interior bodies overlapping with synteny breaks and **b** fractions of TAD intervals overlapping with synteny breaks identified in comparisons of *G. kirkii* *vs.* *G. arboreum* and *G. kirkii* *vs.* *G. raimondii* are not statistically different than those in multiple randomization controls, which involve groups of shuffled TAD interior bodies/intervals (identified synteny breaks are maintained), shuffled synteny breaks (identified TAD interior bodies/intervals are maintained), and both TAD interior bodies/intervals and synteny breaks shuffled simultaneously. The *p* value of Fisher’s two-tailed test in comparison with control is greater than 0.05.


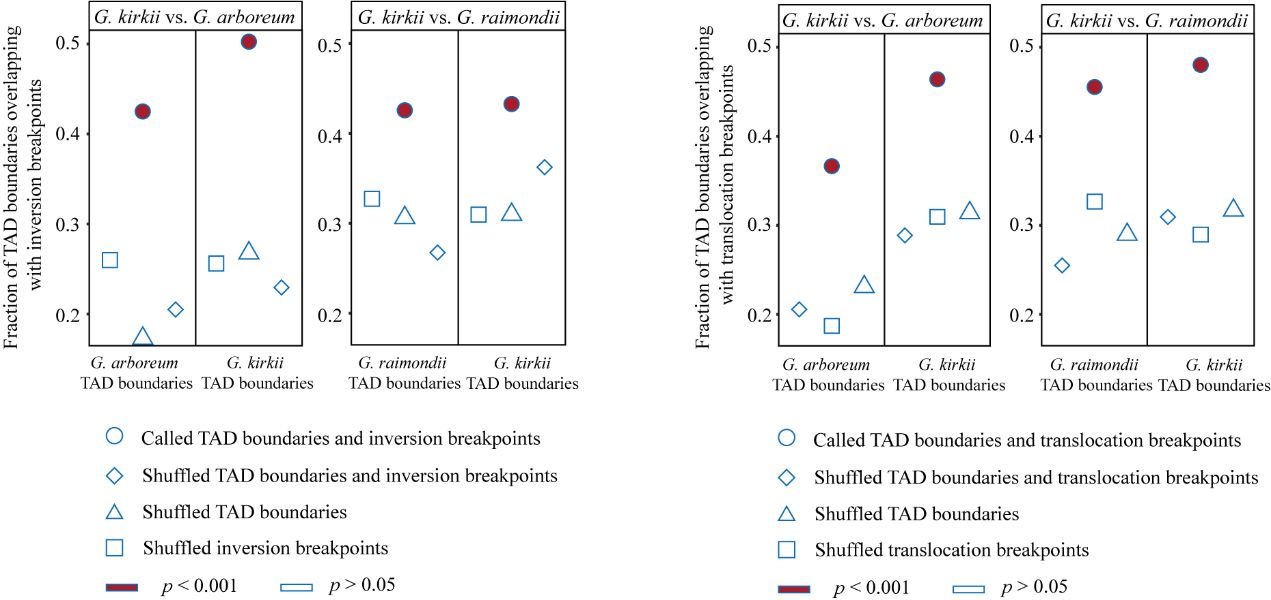


**Figure S12. Fractions of TAD boundaries overlapping with breakpoints of inversion and translocation identified in comparisons of *G. kirkii vs. G. arboreum* and *G. kirkii vs. G. raimondii* are statistically higher than those randomization controls, which involve groups of shuffled TAD boundaries (identified breakpoints are maintained), shuffled breakpoints (identified TAD boundaries are maintained), and both TAD boundaries and breakpoints shuffled simultaneously. The maximum *p* value of Fisher’s two-tailed test in comparison to respective control is still less than 0.001.**


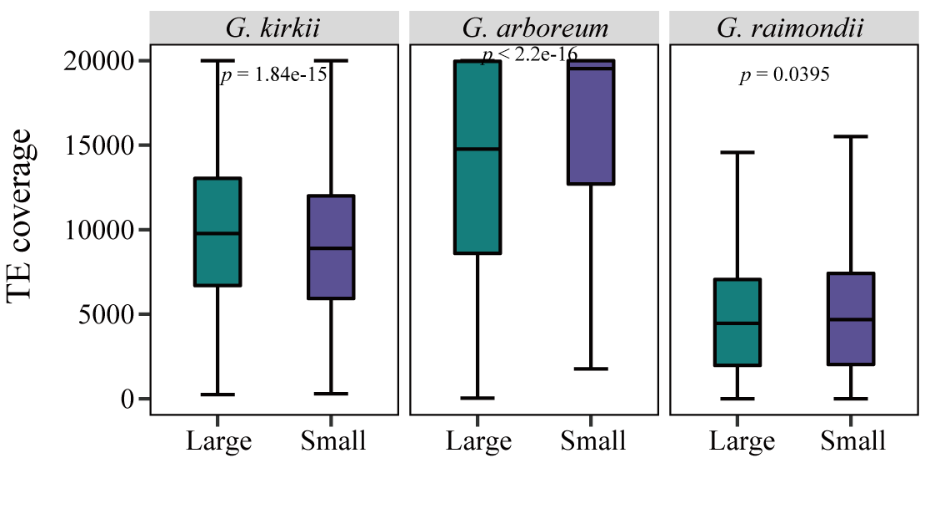


**Figure S13. Abundance of transposable element (TEs) in boundaries of TAD groups (large and small TADs) in *G. kirkii*, *G. arboreum*, and *G. raimondii*, respectively. Statistical significance was calculated using Wilcoxon’s rank sum test.**


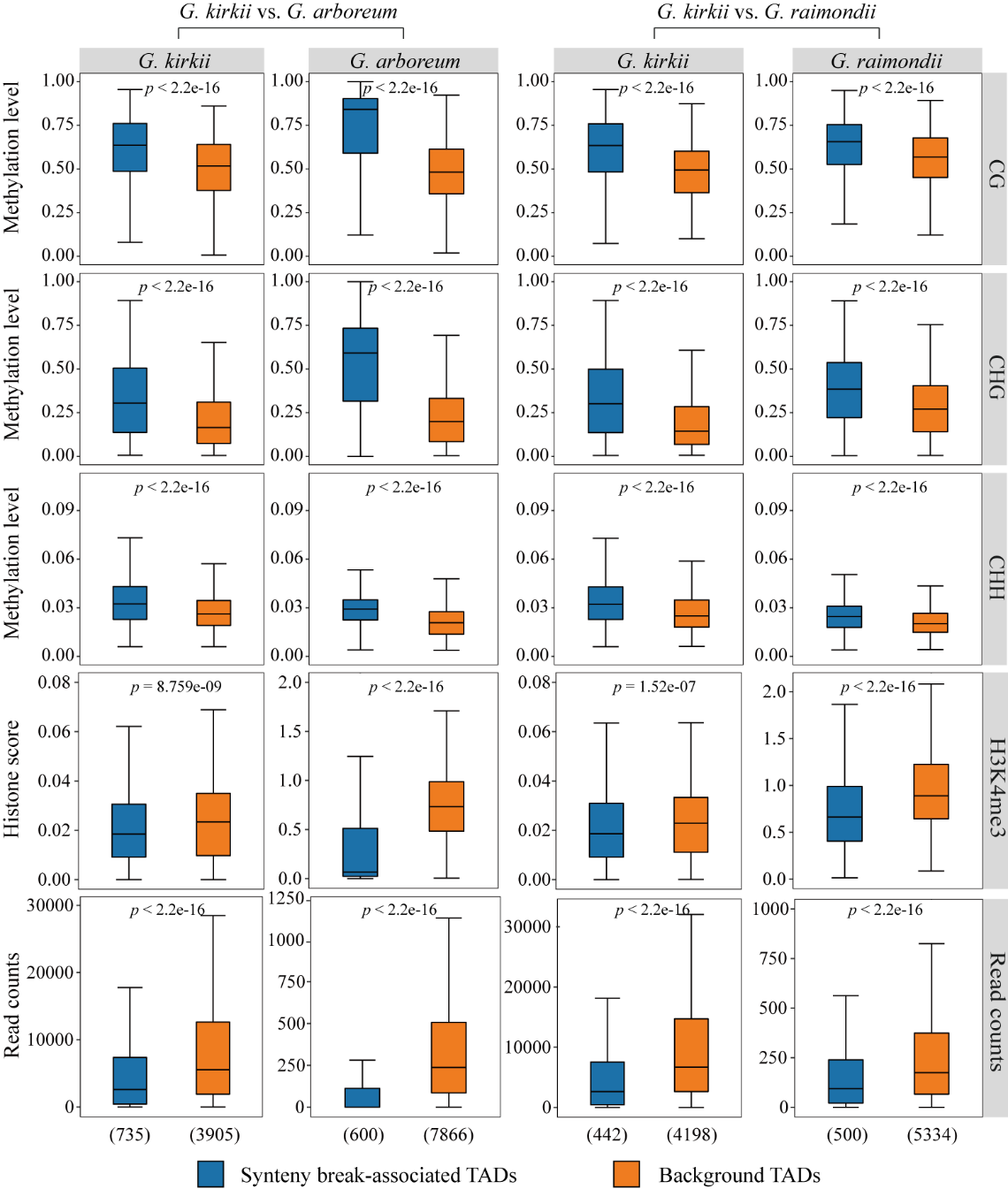


**Figure S14. Open-chromatin epigenetic and active transcriptional features of TAD boundaries co-localizing with synteny breaks.** From top to bottom, DNA methylation level (in CG, CHG, and CHH contexts), abundance of H3K4me3 histone marks, and transcription level at the boundaries of TAD co-localizing with synteny breaks (synteny break-associated TADs) and those of other genomic TADs (background TADs) are summarized in respective species in comparison of *G. kirkii* *vs.* *G. arboreum* and *G. kirkii* *vs.* *G. raimondii*, respectively. The TAD number of two categories of each sample is shown in the parentheses at the bottom. Statistical significance was calculated using Wilcoxon’s rank sum test.


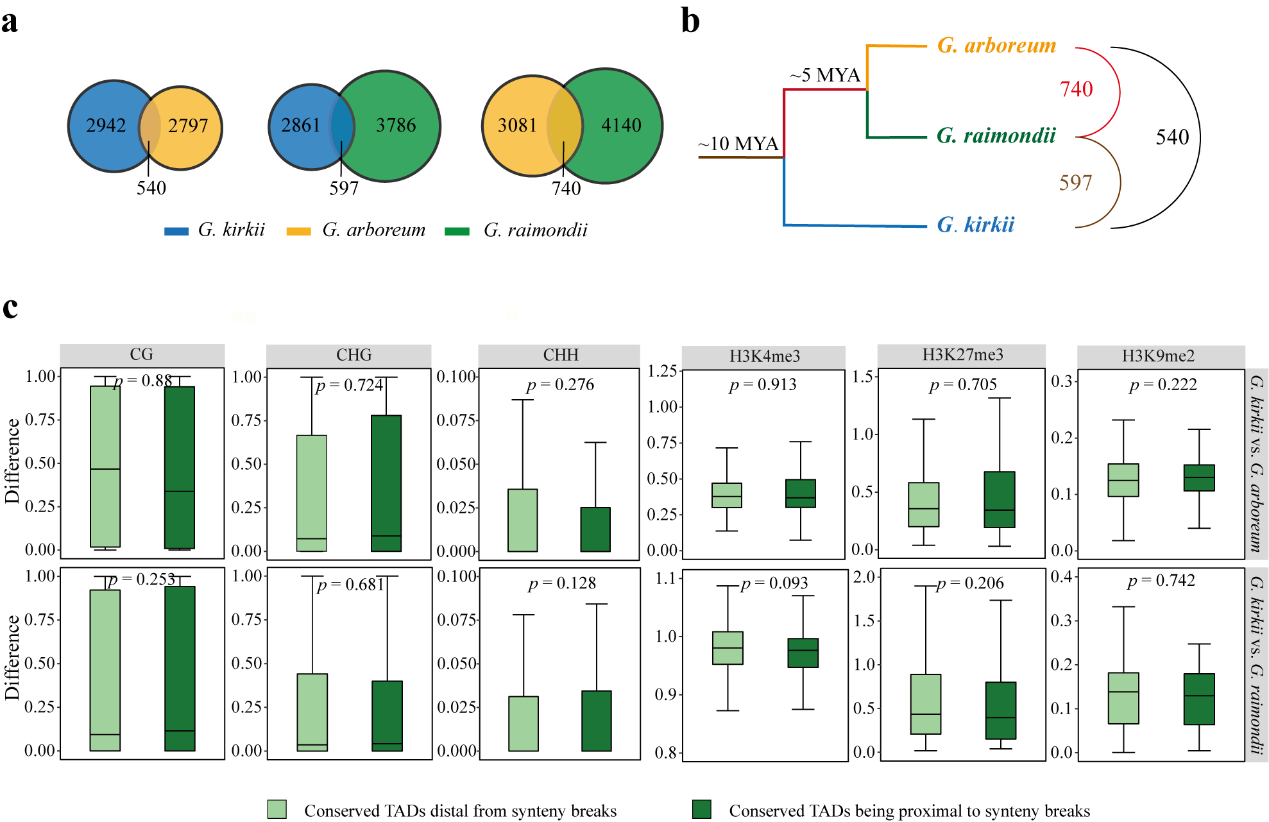


**Figure S15**. **The relationships between genomic rearrangements and epigenetic modifications of syntenic genes for conserved TADs.** **a** Number of conserved TADs identified in *G. kirkii* *vs.* *G. arboreum*, *G. kirkii* *vs.* *G. raimondii*, and *G. arboreum* *vs.* *G. raimondii*, respectively. **b** Number of conserved TADs shared in paired species in a phylogenetic context. **c** Compositional epigenetic modifications (DNA methylation and active/silencing histone modifications) of syntenic gene orthologs in conserved TADs being proximal to synteny breaks *vs.* those being distal from synteny breaks. All statistical significance *p* values were calculated using Wilcoxon’s rank sum test.

**Table S1. Summary of clean Hi-C reads.**

| Sample* | *G. arboreum* | *G. raimondii* | *G. kirkii* |
| --- | --- | --- | --- |
| Read Length (bp) | 100 | 100 | 100 |
| Raw Paired-end Reads | 18,178,162,209 | 8,360,066,895 | 3,036,609,274 |
| Raw Bases (bp) | 5,453,448,662,700 | 2,508,020,068,500 | 910,982,782,200 |
| Clean Paired-end Reads | 17,047,476,314 | 7,906,379,985 | 2,848,392,496 |
| Clean Paired-end Reads Rate (%) | 93.78 | 94.57 | 93.80 |
| Low-quality Paired-end Reads | 473,178,339 | 234,225,079 | 99,957,370 |
| Low-quality Paired-end Reads Rate (%) | 2.60 | 2.80 | 3.29 |
| Ns Paired-end Reads | 9,606,610 | 3,311,691 | 1,050,323 |
| Ns Paired-end Reads Rate (%) | 0.05 | 0.04 | 0.03 |
| Adapter Polluted Paired-end Reads | 647,900,946 | 216,150,140 | 87,209,085 |
| Adapter Polluted Paired-end Reads Rate (%) | 3.56 | 2.50 | 2.87 |
| PolyG Paired-end Reads | 0.00 | 0.00 | 0.00 |
| PolyG Paired-end Reads Rate (%) | 0.00 | 0.00 | 0.00 |
| Raw Q30 Bases Rate (%) | 92.83 | 92 | 92 |
| Clean Q30 Bases Rate (%) | 95.14 | 94.47 | 94.84 |

* Data filtering was completed as follows:

(1) Reads pairs with adapter contamination were removed;

(2) Low quality reads with quality values less than 19 and chimeric reads were filtered. Only reads pairs with mapping quality more than 15 were retained;

(3) Reads with N ratios greater than 5% were discarded (either end of reads with N ratios more than 5%).

**Table S2. Summary of uniquely mapped Hi-C reads.**

| Sample | *G. arboreum* | *G. raimondii* | *G. kirkii* |
| --- | --- | --- | --- |
| Clean Paired-end Reads | 17,047,476,314 | 7,906,379,985 | 2,848,392,496 |
| Unmapped Paired-end Reads | 2,328,276,008 | 653,351,992 | 110,018,317 |
| Unmapped Paired-end Reads Rate (%) | 13.66 | 8.26 | 3.86 |
| Paired-end Reads with Singleton | 5,362,332,923 | 2,160,954,571 | 639,575,837 |
| Paired-end Reads with Singleton Rate (%) | 31.46 | 27.33 | 22.45 |
| Multi Mapped Paired-end Reads | 4,509,620,539 | 1,032,539,236 | 576,301,898 |
| Multi Mapped Ratio (%) | 26.45 | 13.06 | 20.23 |
| Unique Mapped Paired-end Reads | 4,847,246,844 | 4,059,534,186 | 1,522,496,444 |
| Unique Mapped Ratio (%) | 28.43 | 51.35 | 53.45 |

*Clean Hi-C reads were aligned to genome assemblies of *G. arboreum, G. raimondii,* and *G. kirkii* using bowtie2. Subsequently, unmapped reads across the ligation junctions were cut into sub-sequences. The sub-sequences were mapped to the reference genomes again. After combining the output data, only paired-end reads with both mates uniquely mapping to reference genomes (denoted as “Uniquely Mapped Paired-end Reads”) were retained for downstream analysis.

**Table S3. Summary of valid Hi-C reads.**

| Sample | *G. arboreum* | *G. raimondii* | *G. kirkii* |
| --- | --- | --- | --- |
| Unique Mapped Paired-end Reads | 4,847,246,844 | 4,059,534,186 | 1,522,496,444 |
| Dangling End Paired-end Reads | 126,380,818 | 65,415,541 | 8,688,877 |
| Dangling End Rate (%) | 2.61 | 1.61 | 0.57 |
| Self Circle Paired-end Reads | 58,162,034 | 7,781,927 | 4,884,045 |
| Self Circle Rate (%) | 1.20 | 0.19 | 0.32 |
| Dumped Paired-end Reads | 948,997 | 517,795 | 12,173,255 |
| Dumped Rate (%) | 0.02 | 0.01 | 0.80 |
| Interaction Paired-end Reads | 4,661,754,995 | 3,985,818,923 | 1,496,750,267 |
| Interaction Rate (%) | 96.17 | 98.18 | 98.31 |
| Valid Paired-end Reads | 3,738,140,152 | 3,151,068,919 | 1,312,089,687 |
| Valid Rate (%) | 77.12 | 77.62 | 86.18 |

* Invalid uniquely mapped reads as described, such as “Dangling End Paired-end Reads”, “Self Circle Paired-end Reads”, and “Dumped Paired-end Reads” were removed using HiC-Pro. Moreover, we also exclude the invalid reads generated by PCR amplification.

| **Table S4. Pearson correlations of overall chromosomal distributions in *G. kirkii*, *G. arboreum*, and *G. raimondii*.** | | |
| --- | --- | --- |
| Samples | Pearson correlations* | Statistical *p* values |
| *G. kirkii* *vs.* *G. arboreum* | 0.43 | 2.28e-05 |
| *G. kirkii* *vs.* *G. raimondii* | 0.77 | < 2.2e-16 |
| *G. arboreum* *vs.* *G. raimondii* | 0.46 | 4.38e-06 |

* Pearson correlations among inter-chromosomal contact of *G. kirkii* *vs.* *G. arboreum*, *G. kirkii* *vs.* *G. raimondii*, and *G. arboreum* *vs.* *G. raimondii*.

| **Table S5. Epigenetic data from public databases.** | |
| --- | --- |
| Types of data | SRA^*^ |
| *G. arboreum* H3K4me3 for ChIP-seq | SRX3051301 |
| *G. arboreum* H3K27me3 for ChIP-seq | SRX3051300 |
| *G. arboreum* H3K9me2 for ChIP-seq | SRX3051302 |
| *G. raimondii* H3K4me3 for ChIP-seq | SRX3051294 |
| *G. raimondii* H3K27me3 for ChIP-seq | SRX3051293 |
| *G. raimondii* H3K9me2 for ChIP-seq | SRX3051295 |
| ^*^SRA accession number of respective data are tabulated. | |
